# Multiplexed Exchange-PAINT imaging reveals ligand-dependent EGFR and Met interactions in the plasma membrane

**DOI:** 10.1038/s41598-017-12257-y

**Published:** 2017-09-22

**Authors:** Jeffrey L. Werbin, Maier S. Avendaño, Verena Becker, Ralf Jungmann, Peng Yin, Gaudenz Danuser, Peter K. Sorger

**Affiliations:** 1 HMS LINCS Center, Laboratory of Systems Pharmacology, Harvard Medical School, Boston, MA 02115 USA; 2 Department of Systems Biology, Harvard Medical School, Boston, MA 02115 USA; 3 Wyss Institute for Biologically Inspired Engineering, Harvard University, Boston, MA 02115 USA; 4 Department of Cell Biology, Harvard Medical School, Boston, MA 02115 USA; 5 Present Address: Max Planck Institute of Biochemistry and LMU, Munich, Germany; 6 Present Address: Department of Cell Biology, UT Southwestern Medical Center, Dallas, TX 75390 USA

## Abstract

Signal transduction by receptor tyrosine kinases (RTKs) involves complex ligand- and time-dependent changes in conformation and modification state. High resolution structures are available for individual receptors dimers, but less is known about receptor clusters that form in plasma membranes composed of many different RTKs with the potential to interact. We report the use of multiplexed super-resolution imaging (Exchange-PAINT) followed by mean-shift clustering and random forest analysis to measure the precise distributions of five receptor tyrosine kinases (RTKs) from the ErbB, IGF-1R and Met families in breast cancer cells. We find that these receptors are intermixed nonhomogenously on the plasma membrane. Stimulation by EGF does not appear to induce a change in the density of EGFR in local clusters but instead results in formation of EGFR-Met and EGFR-ErbB3 associations; non-canonical EGFR-Met interactions are implicated in resistance to anti-cancer drugs but have not been previously detected in drug-naïve cells.

## Introduction

ErbB receptors comprise a family of four RTKs (EGFR/ErbB1, Her2/ErbB2, Her3/ErbB3 and Her4/ErbB4) with important roles in normal cell physiology and in cancer^1^. ErbB receptors are primarily thought to signal as homo- and heterodimers with other ErbB receptors^2^, but interactions of ErbB receptors with Met^3^ and IGF-1R^4^ RTKs (“non-canonical” interactions) have been reported, particularly in cells that have acquired resistance to ErbB-targeting anti-cancer drugs. Such interactions appear to be weaker than those involving ErbB proteins themselves and have not been detected in drug-naïve cells^3,5^. Immuno-electron microscopy^6^ and fluorescence correlation spectroscopy (FCS)^7^ have shown that ErbB receptors are non-uniformly distributed in the plasma membrane, forming clusters with a characteristic diameter of 100 nm, ~10 times greater than that of an EGFR dimer^8^. Due to limitations in the number of distinguishable immuno-electron probes and FCS-compatible fluorophores, published studies image at most two receptor types at a time^7^.

Single-molecule localization (SML) imaging techniques such as PALM (photoactivated localization microscopy)^9,10^, STORM (stochastic optical reconstruction microscopy)^11,12^ and PAINT (Point Accumulation for Imaging in Nanoscale Topography) and DNA-PAINT^13–15^ achieve resolutions of ~10 nm and make it possible to visualize protein clusters directly. These methods have been applied primarily to highly organized assemblies such as focal adhesions^16^, centrioles^17^, nuclear pore complexes^18^ and the actin cytoskeleton^19^ for which shape and structure are well defined. PALM^20,21^ and STORM^21–23^ have also been employed to image single transmembrane receptors, however the use of SML imaging techniques to map interactions among families of receptors has not yet been reported.

Here we exploit the multiplexing capabilities of Exchange-PAINT, a multiplexed variant of DNA-PAINT^14^, to image simultaneously five RTKs (EGFR, ErbB2, ErbB3, IGF-1R and Met) at endogenous levels of expression in BT20 cancer cells and to examine how receptor distribution changes following ligand stimulation. By using machine learning and subsequent biochemical validation we detect ligand-dependent, non-canonical interaction of EGFR and Met.

## Results

### Validation of Exchange-PAINT imaging of RTKs

**Figure 1:**
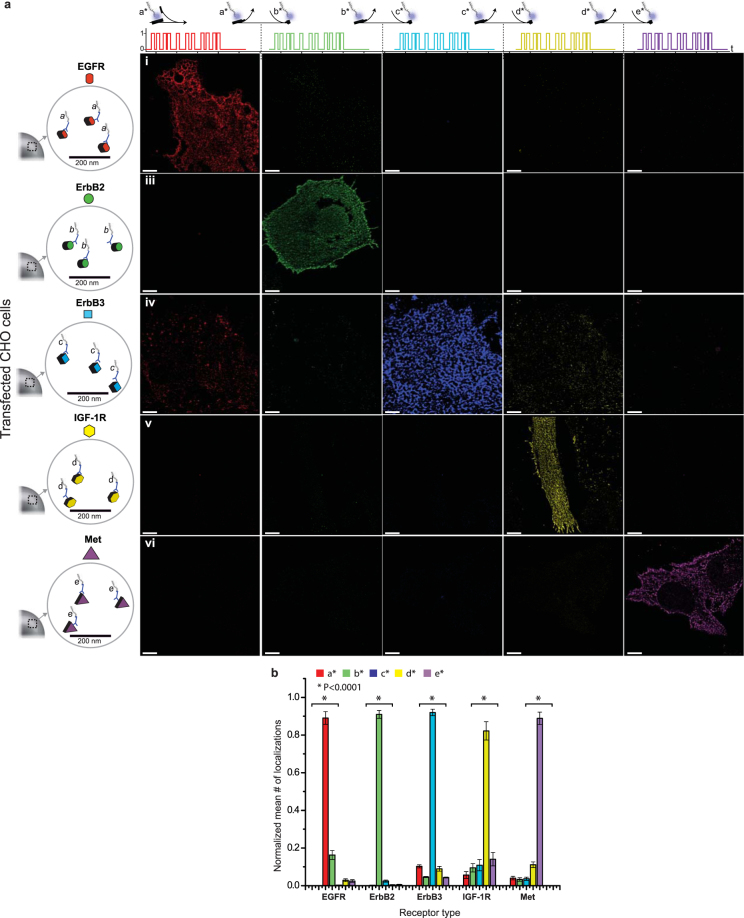
Validation and optimization of Exchange-PAINT for membrane receptor imaging. (**a**) Five CHO cell lines transfected with different human RTKs were fixed, stained and imaged sequentially with 5 rounds of Exchange-PAINT (imager strands at 2.5 nM, 15000 frames, 10 Hz imaging rate). The diagonal images show the specificity of the imaging method and the specificity of the antibody-DNA conjugates for their cognate targets. Scale bars: 5 µm. (**b**) Specificity quantification of images shown in panel a. Localizations were normalized in each group by their maximal number and then the mean was calculated. There are significant differences in the number of localizations for each specifically transfected cell line. *P < 0.0001 ANOVA test, confirming high specificity.

In Exchange-PAINT dye-labelled “imager” strands transiently bind to unlabelled complementary “docking” strands that have been chemically linked to target molecules. Transient binding of the imager strands achieves the ON/OFF switching of fluorescence signals necessary for SML microscopy. The position of each ON/OFF event is referred to as a “localization” and an image is constructed by combining ~10^5^–10^6^ localizations for each probe. By using multiple antibodies each having a unique docking strand it is possible to sequentially image an equivalent number of molecular targets using a single fluorophore and laser source. After completing a round of imaging with one imager strand the sample is washed 5 times until no more localizations are detectable (~1 min) before adding the next imager strand (Fig. 1a).

Selective anti-receptor antibodies compatible with PAINT were identified by testing commercially available reagents on a panel of CHO cell lines each of which transiently over-expressed a single human RTK (CHO express few if any endogenous ErbB receptors^24^) (Fig. 1). Biotinylated monoclonal antibodies were coupled to docking strands with a streptavidin linker^14^ (see online methods and Supplemental Protocol 1), fixed CHO cells were processed by standard immunofluorescence protocols, and fluorescence emission was detected using highly inclined and laminated optical sheet (HILO) microscopy^25^; the use of integrated fluidics made it possible to efficiently perform sequential washing and imaging steps (Supplemental Fig. S1)^14^. For each of the five anti-receptor antibodies in our final collection we observed specificity for the appropriate over-expressing CHO cell line as measured by the total number of localization events (Fig. 1b). To test the reproducibility of the imaging and localization assignment, EGFR was imaged before and after imaging the other four receptors: the two EGFR images had a normalized cross-correlation coefficient of 0.84 demonstrating minimal physical distortion and good sampling of stochastic emissions (Supplemental Fig. S1 **)**.

### Receptor reorganization in response to EGF stimulation

**Figure 2:**
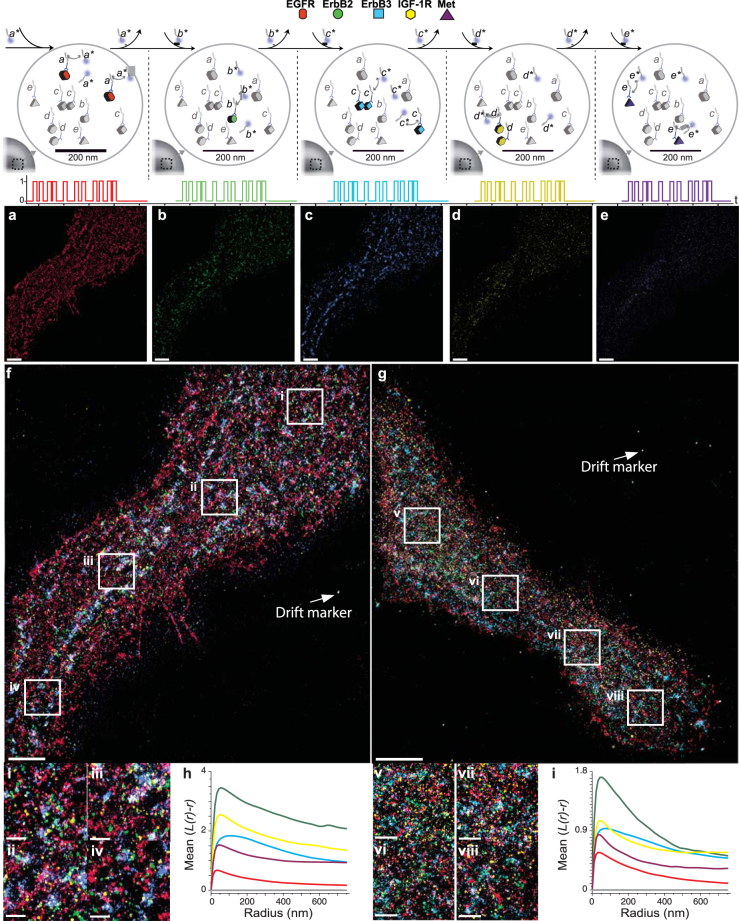
Multiplexed Exchange-PAINT super-resolution imaging of fixed BT20 cells. (**a–e**) Five-“colour” receptor images. (Top) Schematic of imaging procedure. (Bottom) Exchange-PAINT images. Each receptor target is labelled with an antibody carrying a unique Exchange-PAINT docking strand. Imaging is performed sequentially using five orthogonal imager strands (a^*^, b^*^, c^*^, d^*^, e^*^) all at 2.5 nM and labelled with the fluorophore ATTO655. (**f**,**g**) Merged images of all five targets for (**f)** unstimulated or (**g)** EGF-stimulated cells. Inset panels (labelled i to viii) 10-fold higher magnification views of regions of the images in **f** and **g** that highlight receptor clusters with varied shapes and compositions. Imaging: 15,000 frames per cycle at 10 Hz rate. Washing: 1–2 minutes per cycle. (**h**,**i**) Ripley’s K-function analysis of multiple regions selected for unstimulated (**h**) and EGF-stimulated cells (**i**). Mean L(r)—r reports the degree of clustering for several regions in a population of cells relative to a random distribution (indicated by the grey line), and r indicates the radius. Scale bars in **a–g**: 5 µm; scale bars for the insets (labelled i-viii) is 1 µm.

Images of fixed serum-starved (Fig. 2f) and EGF-stimulated (Fig. 2g) BT20 triple negative breast cancer cells, which express all five receptors at detectable levels^26^, were obtained by sequentially imaging the five receptors using the single dye Atto655 (Fig. 2a–e). We detected 2–20 × 10^5^ localizations per cell with an average localization precision of 3.9 nm (representing the single molecule fitting precision) and resulting in a supported resolution of ~9.3 nm (as defined by the full width at half maximum). We also confirmed the localization precision and maximal achievable resolution by comparing the positions of super-localized centres from adjacent frames using the distance between neighbouring-frame localizations (DNFL)^27^. The integral image quality as measured by the Fourier ring correlation method^28^, a metric that combines localization precision, sampling frequency, labeling efficiency and target analyte density into a single measure of effective resolution, was 17.7 ± 3.8 nm (Supplemental Note). Importantly in Exchange-PAINT photobleaching has no significant effect on image acquisition; continuous replenishment of the imaging probe from solution results in a constant number of localizations over the course of image acquisition. (Supplemental Fig. S1).

Images of all five receptors showed that their distribution in the plasma membrane was non-uniform as determined by Ripley’s *K-*function analysis (Fig. 2 insets; see online methods for details)^29^. In both serum-starved and EGF-stimulated cells, stabilized Ripley’s *K*-function curves revealed clustering above that expected for a random distribution (Fig. 2h,i). Receptors of different types were intermingled in both serum-starved and EGF-stimulated cells and there were no obvious patterns corresponding to known receptor homodimers or heterodimers post-stimulation. However, parallel experiments demonstrated strong activation of Erk and Akt signalling kinases, events that require receptor oligomerization^30^, which is consistent with evidence that ErbB receptor dimers can be pre-formed in the absence of ligand^31^. These loose but non-random clumps of RTKs have been previously described^6^ and are thought to have a significant impact on signalling intensity and ligand sensitivity^32^. We hypothesize that EGF exerts its effects on receptors at the level of mesoscopic scale clusters (10–100 nm), but at larger scales RTK distributions are not dramatically changed on short time scales prior to receptor uptake, recycling and degradation (with occurs within 30–90 mins after exposure of BT20 cells to ligand^33^).

### Analyzing spatial patterns using mean-shift clustering

**Figure 3:**
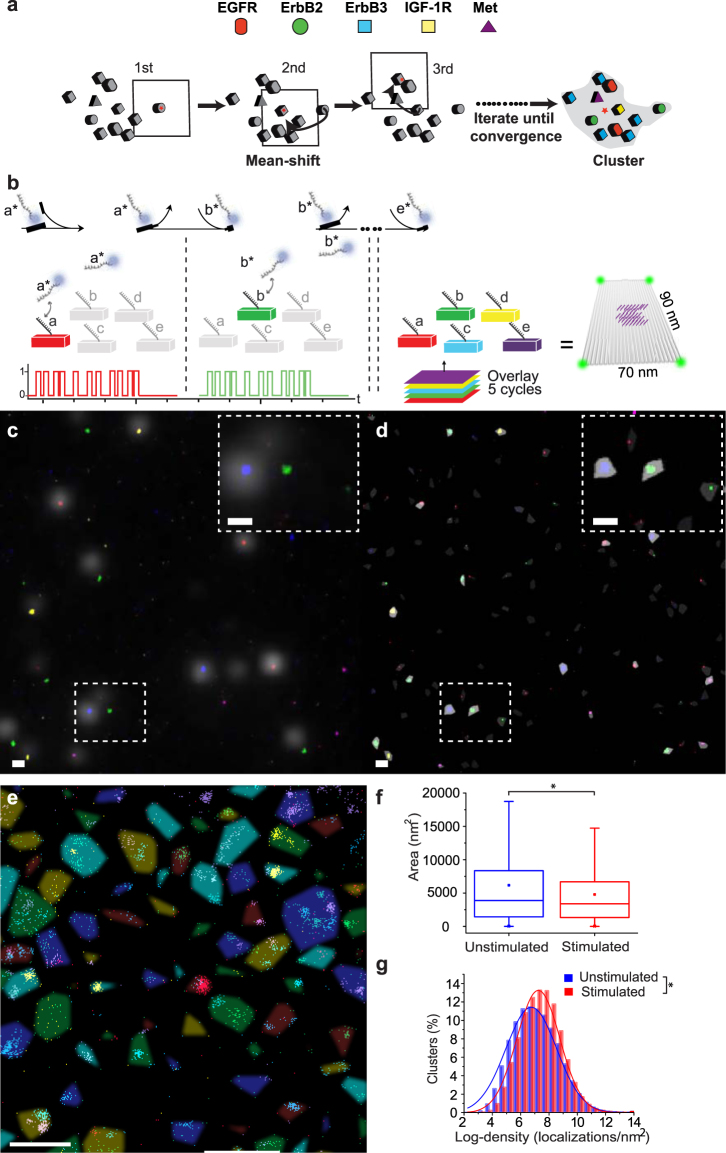
Computational clustering of receptors. (**a**) A schematic of the mean-shift clustering algorithm used to group localizations detected by Exchange-PAINT into clusters based on local density. (**b**) *In vitro* clustering validation. Five types of DNA origami structures (**a** to **e**), each displaying 48 DNA-PAINT docking strands, were mixed and then imaged sequentially as previously described. (**c**) Super-resolved Exchange-PAINT image of the mixed origami sample on a glass surface. Imaging was performed using Cy3b-labelled imager strands at 10 nM, 15,000 frames per cycle, 10 Hz imaging rate. Washing: 1–2 minutes per cycle. Scale bar: 200 nm. (**d**) Localizations from the mixed origami sample in **c** were grouped into clusters (represented in different colours) of high local density as detected by mean-shift clustering. Cluster area is outlined in gray (dashed lines in the upper right corner are 3-fold higher magnification views of outline regions in **c** and **d**. Scale bars: 200 nm. (**e**) Clusters and their outlines in BT20 cells as detected by mean-shift clustering in the image shown in Fig. 2f. Scale bar: 500 nm. (**f**,**g**) Quantitative clustering analysis results after Exchange-PAINT imaging of 5 different receptors comparing unstimulated and EGF-stimulated BT20 cells.

As a means to better quantify receptor distribution in the presence and absence of ligand, localizations were grouped into “clusters” of high local density using mean-shift clustering (Fig. 3a, online methods)^34^. Mean-shift clustering preserves information about the heterogeneity of receptor distribution while capturing information on local density and composition and requires only a single adjustable parameter, the bandwidth (see see online methods for a definition of this term)^35^. Clusters were computed by centering a box, with a width of two times the bandwidth, on each localization and calculating the mean position of all localizations within the box. The box was then shifted to this mean and the process was repeated until convergence (that is, until the mean was stationary). Localizations that converged to the same point were then grouped together and assigned to a cluster. In this analysis we used a fixed bandwidth of 48 nm, which was chosen such that localizations arising from two dimerized receptors would fall within one bandwidth of each other after accounting for experimental uncertainties (see online methods).

The mean-shift clustering approach was tested using DNA origami nanostructures with known binding site number and geometry (Fig. 3b, see Supplemental Fig. S2 for design details). Five origami structures were created, each of which carried 48 copies of one of the docking strands used for receptor imaging. The five structures were mixed in equal proportions and imaged by Exchange-PAINT (Fig. 3c). Following processing and alignment of the images and use of a mean-shift clustering algorithm we observed near-perfect correlation between the original image and the mean-shift clustering result, demonstrating super-resolution discrimination of localizations arising from different probes (Fig. 3d). When mean-shift clustering was applied to Exchange-PAINT data from starved (Fig. 3e), and EGF-treated BT20 cells, we found that clusters in EGF-treated cells were smaller (Fig. 3f) and denser, having a greater number of localizations per unit area (Fig. 3g). This finding is consistent with the results of previous studies that measured ErbB receptor distributions using immunoelectron and scanning near-field optical microscopy^6,7^.

### Machine learning suggests locally driven interactions between Met and EGFR

**Figure 4:**
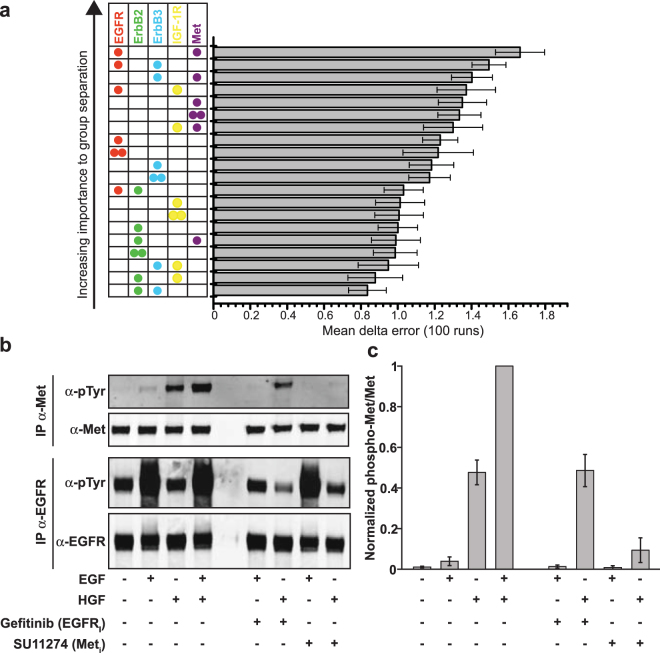
Random forest and biochemical analysis detect cross-stimulation of Met by EGFR following ligand exposure. (**a**) Random forest classification of non-linear combinations of features that reflect molecular interactions between receptors. Feature importance was calculated by repeatedly generating random forests from sub-samples of the data. (**b**) Biochemical analysis of Met-EGFR interaction. Serum-starved BT20 cells were pre-treated for 15 min with 1 µM Gefitinib for EGFR or SU11274 for Met or DMSO as a control and then stimulated for 5 min with EGF (which binds EGFR) and/or HGF (which binds Met) or medium as a control. Receptor immunoprecipitates were subjected to SDS-PAGE and immunoblots were probed with an anti-phospho-tyrosine antibody, anti-EGFR or anti-Met antibodies and respective secondary antibodies. The full length blots are presented in Supplementary Fig. 8. (**c**) Background-subtracted phospho-Met signals were corrected for total Met expression, normalized to the EGF/HGF-treated sample and plotted as mean with standard deviation derived from three biological replicates.

Mean-shift clustering yielded ~5,000–30,000 clusters per BT20 cell (Fig. 3e) each of which was characterized by five features corresponding to individual receptor localization densities. We multiplied local receptor densities with each other to create 15 additional features that represented the likelihood that two receptors were in close proximity within a cluster. To identify which of the resulting 20 features maximally distinguish starved and stimulated cells we used Random Forests (RFs)^36^, a supervised machine learning approach that captures non-linear relationships among heterogeneous types of data. We trained 100 random forests from sub-samples of the data and computed the importance of each of the 20 features in classifying if a cluster was in a starved or EGF- treated cell (Fig. 4a and Supplemental Notes). The forests on average could correctly classify 69.4% (StDev. 0.19%) of the clusters on average. We found that d(EGFR)*d(Met) (where d refers to density within a cluster) was the most significant feature and d(EGFR)*d(ErbB3) was the second most important feature, suggesting that EGFR becomes co-confined with Met and ErbB3 in membrane microdomains following EGF stimulation. EGFR and ErbB3 are known to form functional heterodimers in response to EGF stimulation^37,38^ and Met and EGFR have been reported to interact in cells that have become resistant to anti-ErbB drugs^3,39–41^. Receptor dimers known to mediate the effects of EGF such as EGFR-EGFR and EGFR-ErbB2 were well down the feature importance list, suggesting that the relatively abundant EGFR and ErbB2 receptors are not undergoing significant reorganization at the mesoscopic scale in the time frame of ligand stimulation (5 min). This observation is also consistent with the potential existence of preformed dimers in serum-starved cells^31^.

### Synergistic cross-stimulation of Met by EGFR

As an independent means to determine if EGFR and Met functionally interact in BT20 cells, we immunoprecipitated receptors from starved and ligand-treated cells and examined levels of phosphorylation by immunoblotting of precipitates using a pan-specific anti-phosphotyrosine antibody (Fig. 4b). Compared to unstimulated cells, we observed that Met phosphorylation increased ~4-fold upon exposure of cells to EGF (as compared to ~45-fold upon exposure to the Met-ligand HGF) (Fig. 4c). Concurrent treatment of cells with EGF and HGF was synergistic in promoting Met phosphorylation (Fig. 4c) across a range of ligand concentrations (Supplemental Fig. S3). Conversely, treatment of cells with small-molecule inhibitors targeting EGFR (Gefitinib) or Met (SU11274) prior to EGF addition decreased Met phosphorylation levels, suggesting that both kinases contribute to cross-activation of Met (Fig. 4b,c). Similar results were obtained for inhibitor experiments in cells co-stimulated with EGF and HGF (Supplemental Fig. S4). Thus, Met and EGFR appear to interact at the level of trans-phosphorylation in BT20 cells. However, we were unable to co-immunoprecipitate the two receptors at levels above background (data not shown), suggesting that interactions are low-affinity or indirect^42^.

## Discussion

In this paper we demonstrate the use of multi-target Exchange-PAINT and machine learning to quantify the spatial distribution of five receptors on the plasma membrane of human cells. These receptors were not primarily found in clusters containing a single receptor type but rather were comingled in non-random patches characterized by heterogeneity on multiple length scales. At the outset we had assumed that SML would reveal patterns of receptor co-localization consistent with patterns of receptor homo- and hetero-oligomerization identified by biochemical studies^1,2^. However, the most significant differences between starved and EGF-treated cells involve EGFR-ErbB3 and non-canonical EGFR-Met interactions previously detected in receptor over-expressing cells^5,43^ and implicated in resistance to ErbB-targeting therapies^3^. This implies that ongoing attempts to jointly inhibit ErbB and Met in cancer cells^44^ have a rationale in receptor biophysics. We speculate that relatively low feature importance assigned to d(EGFR) and d(EGFR*EGFR) in the classification of ligand-treated vs. untreated cells is that due to the high abundance of these receptors only minimal changes in their mesoscale densities occur on the time scale of the stimulation, minutes. Our results are also consistent with the presence of preformed dimers or clusters of EGFR.

Exchange-PAINT has several useful properties for studying transmembrane receptors: (1) simplicity of implementation on conventional microscopes, (2) extendibility to additional receptors and multiple cell lines, and (3) ability to identify subtle differences in receptor localization between cell types or cells before and after ligand addition. The availability of antibodies is the primary experimental limitation to the number of receptors that can be imaged simultaneously, but it would theoretically be possible to image all 58 human RTKs with ~10 nm resolution in a native cellular environment.

It is clear that there is much yet to be learned about the organization of RTKs in the plasma membrane and we believe that Exchange-PAINT and machine learning will be potent tools for studying the distribution and interaction of membrane proteins, including those that are being targeted therapeutically using antibody-based drugs. Such drugs are also ideal detection reagents for Exchange-PAINT. With respect to data analysis, we experimented with a number of approaches before choosing mean-shift clustering and random forest algorithms because they were biologically interpretable and well-suited to characterizing complex distributions (Supplemental Notes). However, we anticipate that other statistical techniques will also prove useful in the interpretation of multiplex Exchange-PAINT data. We therefore provide all images and localization data in this paper as supplementary material and at http://lincs.hms.harvard.edu/werbin-sci-rep-2017/.

## Methods

### Materials

Non-modified and amino-modified DNA oligonucleotides were purchased from Integrated DNA Technologies (Coralville, IA). Fluorescently labelled DNA oligonucleotides were purchased from Biosynthesis (Lewisville, TX).

### Plasmids

To clone ErbB receptor-mEOS2 fusion constructs, mEOS2 DNA (a gift from Michael Davidson) with a 5′-GGSGG-3′ linker was inserted into the first variable region of the pQCXIP plasmid (CloneTech) between AgeI and EcoRI. ErbB receptors EGFR, ErbB2, or ErbB3 (a gift from William Hahn & David Root, Addgene plasmids #23935, #23888, and #23874, respectively)^45^ were amplified by PCR amplification and inserted into NotI and AgeI sites of pQCXmEOS2IP (To be deposited at Addgene).

### Cell culture

BT20 cells were obtained from ATCC and cultivated in EMEM (ATCC, Manassas, VA) supplemented with 10% heat-inactivated fetal bovine serum (FBS) (Invitrogen, Carlsbad, CA) and 1% Penicillin-Streptomycin (PS). CHO cells were cultivated in F-12K (ATCC, Manassas, VA) supplemented with 10% FBS and 1% PS and transfected with pQC-EGFR-mEOS2-IP, pQC-ErbB2-mEOS2-IP, pQC-ErbB3-mEOS2-IP, pBabe-IGF-1R (a gift from Ronald Kahn, Addgene plasmid #11212)^46^ or pBabe-Met (a gift from Joan Brugge, Addgene plasmid #17493)^47^ using TurboFect (Thermo Scientific, Waltham, MA).

### Super-resolution optical setup

Fluorescence imaging was carried out on an inverted Nikon Eclipse Ti microscope (Nikon Instruments, Melville, NY) with the Perfect Focus System, applying an objective-type TIRF configuration using a Nikon TIRF illuminator with an oil-immersion objective (CFI Apo TIRF 100× , NA 1.49, Oil), corresponding to a final pixel size of 160 nm. Two lasers were used for excitation: 561 nm (200 mW nominal, Coherent Sapphire) (for diffraction limited imaging) and 647 nm (300 mW nominal, MBP Communications, Canada) (for Exchange-PAINT). The laser beam was passed through cleanup filters (ZET561/10, and ZET640/20, Chroma Technology, Bellows Falls, VT) and coupled into the microscope objective using a multi-band beam splitter (ZT561rdc/ZT640rdc, Chroma Technology). Fluorescence light was spectrally filtered with emission filters (ET600/50 m, and ET700/75 m, Chroma Technology) and imaged on an EMCCD camera (iXon X 3 DU-897, Andor Technologies, North Ireland).

### Antibody-DNA conjugates

Antibody-DNA conjugates used to specifically label receptors with DNA-PAINT docking sites were preassembled in three steps. Three buffers were used for sample preparation and imaging: Buffer A (10 mM Tris-HCl, 100 mM NaCl, 0.05% Tween-20, pH 7.5), buffer B (5 mM Tris-HCl, 10 mM MgCl_2_, 1 mM EDTA, 0.05% Tween-20, pH 8), and buffer C (1× PBS, 500 mM NaCl, pH 8).

#### Labelling streptavidin

Streptavidin (Cat. No. 43-4302, Invitrogen, Carlsbad, CA) was mixed with biotinylated oligo (bt-oligo) (Integrated DNA Technologies, Coralville, IA) at a 1:3 molar ratio and incubated for 30 min at room temperature. This maximizes the amount of the streptavidin in the 3 × (bt-oligo) state; ideal amount is ~40% as estimated by the binomial distribution.

#### Separating triply oligo bound streptavidin

A GE MonoQ 5/50 was cleaned with 1 ml of 2 M NaCl and then equilibrated with 15 ml of buffer 1 (20 mM Tris pH 6.3, 300 mM NaCl). The streptavidin-oligo reaction was brought to 20 mM Tris pH 6.3 and then diluted to 400 µl with buffer 1 before being applied to the column. 5 ml of buffer 1 was run on the column at a flow rate of 0.2 ml/min, and then a linear gradient between 20% buffer 2 (20 mM Tris pH 6.3, 1 M NaCl) and 40–65% (depending on the oligo) over the course of 25 min. Unlabelled streptavidin does not bind to the column and the single, double, triple and quadruple labelled streptavidin came off the column in well separated peaks (Supplemental Fig. S5). The triply labelled fractions were collected and brought to ~100 mM Tris-HCl pH 7.4. *Labelling biotinylated antibodies*. Biotinylated monoclonal antibodies against EGFR (Cat. No. 6627, Cell Signaling, Danvers, MA), ErbB2 (custom biotinylation of clone 242D, BioLegend, San Diego, CA), ErbB3 (Cat. No. 324704, BioLegend, San Diego, CA), IGF-1R (Cat. No. MA5-13799, Thermo Scientific, Waltham, MA) or Met (Cat. No. 8041, Cell Signaling, Danvers, MA) were mixed with the triply labelled streptavidin at a 1:2 molar ratio, incubated at room temperature for 30 min and then concentrated using a 10 kDa cutoff Amicon 4 ml spin concentrator (UFC801024, Millipore, Billerica, MA) until the antibody concentration was >1 mg/ml. Concentrated antibody conjugates were diluted to a final concentration of 0.5 mg/ml for EGFR, ErbB2, ErbB3 and IGF-1R or 0.085 mg/ml for Met in ~35 mM Tris pH 7.4, 300 mM NaCl, 1 mg/ml BSA, 0.2% NaAzide, 50% Glycerol and stored at −20 °C.

### Sample preparation, acquisition, and analysis for Exchange-PAINT imaging of BT20 and CHO cells

#### Coated Lab-Tek II chambers

8-well Lab-Tek II chambered coverglass (Cat. No. 155409, Thermo Scientific, Waltham, MA) were coated with fibronectin (Cat. No. F1141, Sigma Aldrich, St. Louis, MO) at a concentration of 10 µg/ml FN in PBS (containing Ca and Mg) for 1 hour at 37 °C, then washed with PBS.

#### Sample preparation

BT20 cells were seeded in coated 8-well Lab-Tek II chambered coverglass, washed twice and starved overnight in serum-free medium (EMEM). One hour prior to stimulation, cells were re-starved to remove autocrine ligands and then exposed to EGF (Peprotech, Princeton, NJ) at saturating concentration (10 µg/mL) for 5 min or to medium alone as a control. Stimulated and starved cells were immunostained using the following procedure: fixation with 4% paraformaldehyde for 30 min; washing twice with TBS; reduction with 1 mg/ml NaBH_4_ for 7 min; washing three times with TBS; permeabilization and blocking with blocking buffer (0.3% (v/v) Triton X-100 and 3% BSA in TBS) for at least 2 hours; blocking endogenous avidin and biotin (Cat. No. 00-4303, Invitrogen, Carlsbad, CA) and staining overnight with preassembled antibody-DNA conjugates against EGFR, ErbB2, ErbB3, IGF-1R (diluted to 3 µg/ml in blocking buffer), and Met (diluted to 0.56 µg/ml in blocking buffer) at 4 °C. CHO cells were seeded in coated Lab-Tek II chambered coverglass 24 hours after transfection, starved overnight and fixed and stained as described above.

#### Imaging conditions

A Lab-Tek II chamber was adapted for fluid exchange as shown in Supplemental Fig. S1. For Figs 1 and 2 and Supplemental Fig. S1, 2.5 nM Atto 655-labelled imager strands in buffer C were used. Each image acquisition step was followed with a brief ~1 min washing step consisting of at least five washes using 200 µl of buffer C each. Then the next imager strand solution was introduced. The chamber was monitored throughout the washing procedure to ensure complete exchange of imager solutions. Acquisition and washing steps were repeated until all five receptor targets were imaged. The CCD readout bandwidth was set to 3 MHz at 14 bit and 5.1 pre-amp gain. No EM gain was used. Imaging was performed using highly inclined (HILO) illumination^25^ as described above with an excitation power of ~50 mW and imaging intensity of ~0.2 kW/cm^2^ using the 647 nm laser line.

#### Data analysis

Super-resolution images were reconstructed using spot-finding and 2D-Gaussian fitting algorithms in LabVIEW^13,14^. Analysis programs are available for download at http://www.dna-paint.net or http://molecular-systems.net.

### DNA origami self-assembly

DNA origami structures displaying 48 Exchange-PAINT docking strands were self-assembled in a one-pot reaction with 50 µl total volume containing 10 nM scaffold strand M13mp18 (New England Biolabs, Ipswich, MA), 100 nM folding staples, 100 nM biotinylated staples, and 1 µM DNA-PAINT docking staple strands in folding buffer (1 × TE Buffer with 12.5 mM MgCl_2_). The solution was annealed using a thermal ramp cooling from 90 °C to 20 °C over the course of 1.5 h. After self-assembly, structures were purified by agarose gel electrophoresis (2% agarose, 0.5 × TBE, 10 mM MgCl_2_, 0.5 × SybrSafe) at 4.5 V/cm for 1.5 h. Gel bands were cut, crushed and filled into a Freeze ‘N Squeeze column (Bio-Rad, Hercules, CA) and spun for 5 min at 800 × g at 4 °C. After this, structures were ready for preparation as microscope samples followed by image acquisition.

### DNA sequences

Staple sequences for DNA origami structures with 48 docking strands and fixed Cy3 dyes can be found in Supplemental Table S1. M13mp18 scaffold sequence for DNA origami structures can be found in Supplemental Table S2. Exchange-PAINT docking and imager strands sequences and biotin docking strands sequence can be found in Supplemental Table S3.

### Sample preparation, imaging, and analysis of DNA origami structures

#### Sample preparation

For fluid exchange, a custom flow chamber was constructed similar as in cellular imaging. For binding of the origami structures to the surface of the flow chamber first, 20 µl of biotin-labelled bovine albumin (Sigma Aldrich, St. Louis, MO; 1 mg/ml, in buffer A) was applied to the chamber and incubated for 2 min. The chamber was then washed using 40 µl of buffer A. 20 µl of streptavidin (0.5 mg/ml, in buffer A) was then flown through the chamber and allowed to bind for 2 min. After washing with 40 µl of buffer A and subsequently with 40 µl of buffer B, 20 µl of biotin-labelled DNA structures (~300 pM) in buffer B were finally flown into the chamber and incubated for 5 min. The chamber was washed using 40 µl of buffer B.

#### Imaging conditions

Sequential imaging was done as described for BT20 cell imaging, but the washing steps were performed using buffer B. The imaging buffer contained 10 nM Atto 655-labelled imager strands in buffer B (Fig. 3c). Acquisition and washing steps were repeated until all five origami structures were imaged. The CCD readout bandwidth was set to 3 MHz at 14 bit and 5.1 pre-amp gain. No EM gain was used. Imaging was performed using TIRF^25^ illumination with an excitation power of ~50 mW and an imaging intensity of ~0.2 kW/cm^2^ using the 647 nm laser line.

#### Data analysis

Super-resolution images were reconstructed as described before.

### Drift correction

#### *In vitro* imaging

Drift correction was performed using a custom-written MATLAB program. The positions of all DNA origami structures was tracked throughout the duration of each movie and averaged for use as the drift correction trace.

#### *In situ* imaging

For cellular imaging, 100 nm gold nanoparticles (Sigma Aldrich, St. Louis, MO; 10 nM in buffer C, added before imaging) were used as drift markers. The gold nanoparticles adsorb non-specifically to the glass bottom of the imaging chambers. Drift correction is performed in a similar fashion as for the *in vitro* imaging (see above). The apparent “movement” of all gold nanoparticles in a field of view is tracked throughout the movie. The trajectories thus obtained are averaged and used for global drift correction of the final super-resolution image.

#### Image alignment

All single colour images are aligned by fitting a 2D Gaussian to the common drift markers and finding the *x*-*y* translation that best aligns the markers to the IGF-1R image. This translation is applied to all localizations before further analysis.

### Ripley’s K analysis

A modified version of Ripley’s K function was used to analyze the localization images for each channel separately. The modified version of the Ripley’s statistic here referred to as L(r)-r is a transformation of the K(r) function so that random point distributions produce zero response at all spatial scales. To prevent edge artefacts L(r) was calculated for several small rectangular regions of interest (ROIs) from within each cell such that the edge of the cell was not included in the ROI.

1 $${\rm{K}}({\rm{r}})=\frac{A}{n}\sum _{i}\sum _{j}I({d}_{ij} < r)$$

2 $$L(r)=\sqrt{\frac{K(r)}{\pi }}$$

Here, *n* is the number of points contained within the ROI but are at least a distance of *r* from the edge, *dij* is the distance between two points *i* and *j*, *r* is the analyzed spatial scale, and *I* is an indication function with a value of 1 if the statement is true and 0 if false. This function is a measure of the number of points, *j*, enclosed in concentric circles of radius *r* centred on each point *i*. The K-function scales with circle area for a completely spatially random case, and *L* scales linearly with area. For ease of interpretation we plot *L(r)-r* vs *r* which has an expected value of 0 for all *r* in the case of randomly distributed points (see Fig. 2).

### Mean-shift clustering analysis

Localizations from 5 images were merged into a single list and mean shift clustering was applied with a 48 nm bandwidth. The bandwidth is defined as half of the width of the square window used in Mean-Shift clustering. Thus when the window is centred on a localization the bandwidth determines the furthest distance to another localization that will be considered to potentially belong to the same cluster. Here the bandwidth was chosen from first principles such that if two receptors are dimerized any localizations from bound antibodies will fit within the analysis window simultaneously. By way of illustration consider the case in which localizations arising from binding of two oligo-coupled antibodies to a single receptor dimer are as far apart as possible in the imaging plane. In this case a) antibodies bind to the receptors as far as possible from the dimerization interface placing the two antibodies ~8 nm from each other^48^, b) the antibodies lie in the plane of the membrane and point away from each other (10 nm per antibody)^49^. We must also consider c) the distance between biotin binding sites of ~3 nm^50^, d) the length of 10 bp ssDNA of ~3 nm^51^ and e) the localization uncertainty from fitting the dyes of approximately 4 nm per dye. When all of these uncertainties are added together we obtained 20 nm for each antibody + 8 nm for the receptors so that localizations from a single receptor dimer could be appear to be as far apart as 48 nm. We therefore used this distance as the bandwidth parameter for mean-shift clustering. The convex hull of each cluster was calculated and used to define the area of the cluster. We defined the composition as the number of localizations of each receptor type within the cluster and the densities as the number of localization divided by the cluster area in nm^2^. If a cluster had fewer than 12 localizations in total it was discarded because it is likely to be noise. This cut-off is derived from considering that a single bound antibody should yield ~43 localizations (see below for justification). Only clusters that were internal to a cell and at least 100 nm from either the cell or image boundary were kept for further analysis. The cluster densities of *n* = 11 unstimulated (serum-starved) cells were compiled into an *n* cluster by 5-feature array, where the features are the localization densities for each receptor type. The same procedure was applied to *n* = 11 EGF stimulated cells.

#### Rationale for excluding cluster with fewer than 12 localizations

Each antibody is labelled on average with 6 DNA oligo binding strands as determined by FPLC in Supplemental Fig. S5. At a concentration of 2.5 nM imager strands an average of 7.2 localizations^13^ is expected per target strand while imaging a single “channel”. The smallest real cluster must contain at least one antibody and at least one streptavidin molecule with 6 target strands. Assuming that localizations are distributed according to a Poisson process with a rate of λ = (7.2 localizations/(imaging period*strand)) * 6 strands = 43.2 localizations/imaging period, the probability of an antibody having producing exactly 12 localizations is given by ${\lambda }^{12}{e}^{-\lambda }/12!$ ~1.5 × 10^−8^ and the probability of 12 or fewer localization is given by ${e}^{-\lambda }\ast \sum _{n=0}^{12}{\lambda }^{n}/n!$ ~ 2 × 10^−8^. Given that ~2 × 10^5^ clusters were observed before applying this cut-off, it is unlikely that any localizations arising from a real antibody were discarded.

### Random forest analysis

The family of RTKs tested in this work requires formation of dimers for signalling. Thus, in addition to examining receptor densities, we constructed features for each cluster describing the coupling of receptor densities. This was accomplished by pairwise multiplication of the individual receptor localization densities (features), creating 15 additional features for each cluster (since multiplication is commutative there are only 15 unique features of the form feature_i_* feature_j_). Our training matrix was therefore of the form (n_unstimulatedClusters_ + m_unstimulatedClusters_) × 20 features. Each cluster was labelled with either 0 or 1 to indicate that the cluster was from an unstimulated or stimulated cell respectively. This data was used to generate a random forest using the Matlab function treeBagger which is part of the Matlab Statistics and Machine Learning toolbox. All forests in this paper were grown with 50 trees (NTrees = 50), with a minimum number of observations per leaf of 300 (MinLeaf = 300) and with variable importance prediction turned on.

We then calculated 100 random forests from random sub-samples of our data. This allowed us to remove class imbalance and estimate uncertainty of the variable importance measure. The sub-samples were generated by randomly selecting 66% of the unstimulated data and then randomly selecting an equal amount of stimulated data. From this data a random forest was trained and the variable importance (mean delta error) for each feature was recorded.

The variable importance of the *i* ^*th*^ feature was calculated by taking the out of bag (OOB) data for each tree in the forest and determining the percentage of correct classification, ErrorOOB. Then, the *i* ^*th*^ feature of the OOB data was scrambled and the classification error Error_i^th^Random calculated. The delta error was defined as ErrorOOB – Error_i^th^Random. The reported mean delta error is the mean of this number over all trees in the forest. This number was recorded for all 20 features for each sub-sampling of the data. The results are presented in Fig. 4a.

### Sample preparation and analysis of immunoprecipitates

To prepare detergent lysates, BT20 cells were seeded in 60 mm-dishes or 100 mm-dishes, washed twice and starved for 15–18 hours in serum-free medium (EMEM). One hour prior to the start of the experiment, cells were re-starved to remove autocrine ligands. If not otherwise indicated, cells were pre-treated for 15 min at 37 °C with 1 µM Gefitinib or SU11274 (Selleck Chemicals, Houston, TX) or with DMSO as a control and subsequently stimulated for 5 min at 37 °C with EGF (10 µg/ml), HGF (300 ng/ml), both ligands together or culture medium as a control (ligands from Peprotech, Princeton, NJ). Cells were transferred to ice, washed with ice-cold PBS and lysed with a 1% NP40 lysis buffer (1% NP40, 150 mM NaCl, 20 mM Tris pH 7.5, 10 mM NaF, 1 mM EDTA pH 8.0, 1 mM ZnCl_2_ pH 4.0, 1 mM MgCl_2_, 1 mM Na_3_VO_4_, 10% Glycerol) supplemented with Complete mini/EDTA-free protease inhibitors (Roche, Penzberg, Germany). Cleared lysates were subjected to immunoprecipitation by incubating samples with rabbit antibodies against EGFR (Cat. No. 6627, Cell Signaling, Danvers, MA) or Met (Cat. No. sc-10, Santa Cruz, Dallas, TX) and Protein A-coupled Dynabeads (Invitrogen, Carlsbad, CA). Immunoprecipitates were washed twice with 1% NP40 lysis buffer and once with TNE buffer (10 mM Tris pH 7.5, 100 mM NaCl, 1 mM EDTA pH 8.0, 100 µM Na_3_VO_4_), resuspended in 1 × NuPage LDS Sample Buffer supplemented with 50 mM DTT, boiled for 3 min at 95 °C, and loaded on Novex 3–8% Tris-Acetate gels (Invitrogen, Carlsbad, CA). Immunoblots were performed using the iBlot Gel Transfer Stacks PVDF system (Invitrogen, Carlsbad, CA). After blocking with Odyssey Blocking Buffer (Licor, Lincoln, NE), membranes were incubated with primary (mouse anti-phosphotyrosine antibodies, Cat. No. 05-321, Millipore, Billerica, MA; rabbit anti-EGFR antibodies, Cat. No. 4267, Cell Signaling, Danvers, MA; rabbit anti-Met antibodies, Cat. No. 8041, Cell Signaling, Danvers, MA) and secondary antibodies (anti-mouse antibodies coupled to DyLight 680, Cat. No. 35518 or anti-rabbit antibodies coupled to DyLight 800, Cat. No. 35571, from Thermo Scientific, Waltham, MA) diluted in Odyssey Blocking Buffer. Membranes were scanned on a Licor Odyssey CLx scanner using the 700 and 800 nm channels set to automatic intensity with a 169 µm resolution. Protein levels were quantified with the Licor Image Studio 4.0.21 software using the build-in manual analysis tool with a median local background correction. Intensity values for phospho-Met were divided by intensity values for total Met expression from the same blot and were subsequently normalized to the EGF/HGF-stimulated sample. Relative protein levels between conditions (phosphorylated and total receptor) were derived from the same blot.

## Electronic supplementary material

Supplementary Information
