# Supplementary material for: Multiplexed Exchange-PAINT imaging reveals ligand-dependent EGFR and Met interactions in the plasma membrane
Source: Sci Rep. 2017 Sep 22;7:12150. doi: 10.1038/s41598-017-12257-y (PMC5610318; doi:10.1038/s41598-017-12257-y)
Supplement: Supplementary file 1 — Supplementary Information [file 41598_2017_12257_MOESM1_ESM.pdf]

Multiplexed Exchange-PAINT imaging reveals ligand-dependent EGFR and Met interactions in the plasma membrane

Jeffrey L Werbin, Maier S Avendaño, Verena Becker, Ralf Jungmann, Peng Yin, Gaudenz Danuser & Peter K Sorger

**Supplemental Figure S1**	**Exchange-PAINT setup and performance**
**Supplemental Figure S2**	**Strand diagram and schematics for DNA origami structures**
**Supplemental Figure S3**	**Cross-stimulation of Met by EGFR across a range of ligand concentrations**
**Supplemental Figure S4**	**Inhibitor experiments in cells co-stimulated with EGF and HGF**
**Supplemental Figure S5**	**Separation of biotinylated oligo-streptavidin conjugates by FPLC and characterization by native gel electrophoresis**
**Supplemental Figure S6**	**Alternative analysis for comparing unstimulated to EGF stimulated receptor clustering data**
**Supplemental Figure S7**	**Resolution measured by Fourier Ring Correlation and Distance between Neighbouring-Frame Localizations**
**Supplemental Figure S8**	**Full-length blots from Figure 4**
**Supplemental Table S1**	**Staple sequences for 48 docking strands origami with fixed Cy3 dyes**
**Supplemental Table S2**	**M13mp18 scaffold sequence for DNA origami structures**
**Supplemental Table S3**	**DNA-PAINT docking and imager sequences and biotin docking sequence**
**Supplemental Protocol 1**	**Antibody-DNA conjugates**
**Supplemental Protocol 2**	**Measuring receptor clustering distortion during Exchange-PAINT imaging**
**Supplemental Note**	**Rationale for choosing Random Forests analysis**
**Supplemental Note**	**Dissociation rate of streptavidin-biotin bond**
**Supplemental Note**	**Rationale for EGF stimulation time of 5 min**
**Supplemental Note**	**Fourier ring correlation**

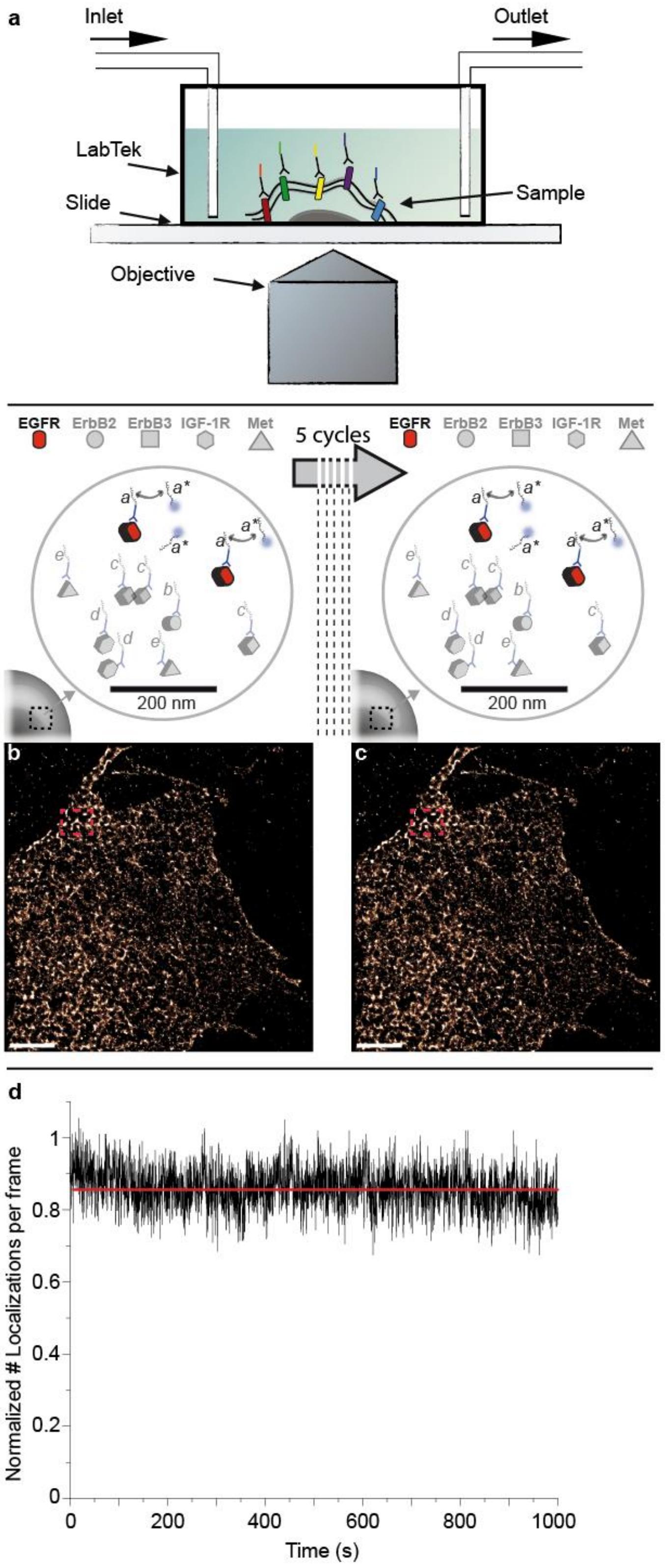

Supplemental Figure 1: Exchange-PAINT setup and performance. (**a**) **Experimental setup used for cell imaging.** Cells were imaged in a coated Lab-Tek II chambered coverglass. One syringe supplied new buffer solution (inlet), the second one the buffer (outlet). (**b**) **Quantification of receptor clustering distortion during Exchange-PAINT imaging.** EGFR imaging was performed twice, at the beginning and again after 5 rounds of Exchange-PAINT imaging, allowing quantification of possible clustering distortion after several washing steps. (**b**) First, EGFR imaging was performed using imager sequence a*. (**c**) After 5 rounds of Exchange-PAINT with multiple washing steps, EGFR was imaged again. The same region of interest (ROI), highlighted by a red rectangle in **b** and **c** was selected in each of the two images. The cross-correlation coefficient was determined to be 0.8348. **(d) Constant number of localizations during acquisition time.** The number of single-molecule localization events remained constant over time during Exchange-PAINT imaging, demonstrating that this technique is not compromised by “photobleaching”. Black: localizations per frame; red: zero-slope (constant) linear curve as a guide to the eye. Data obtained from image shown in **Fig. 1a**. Scale bars: 5 µm

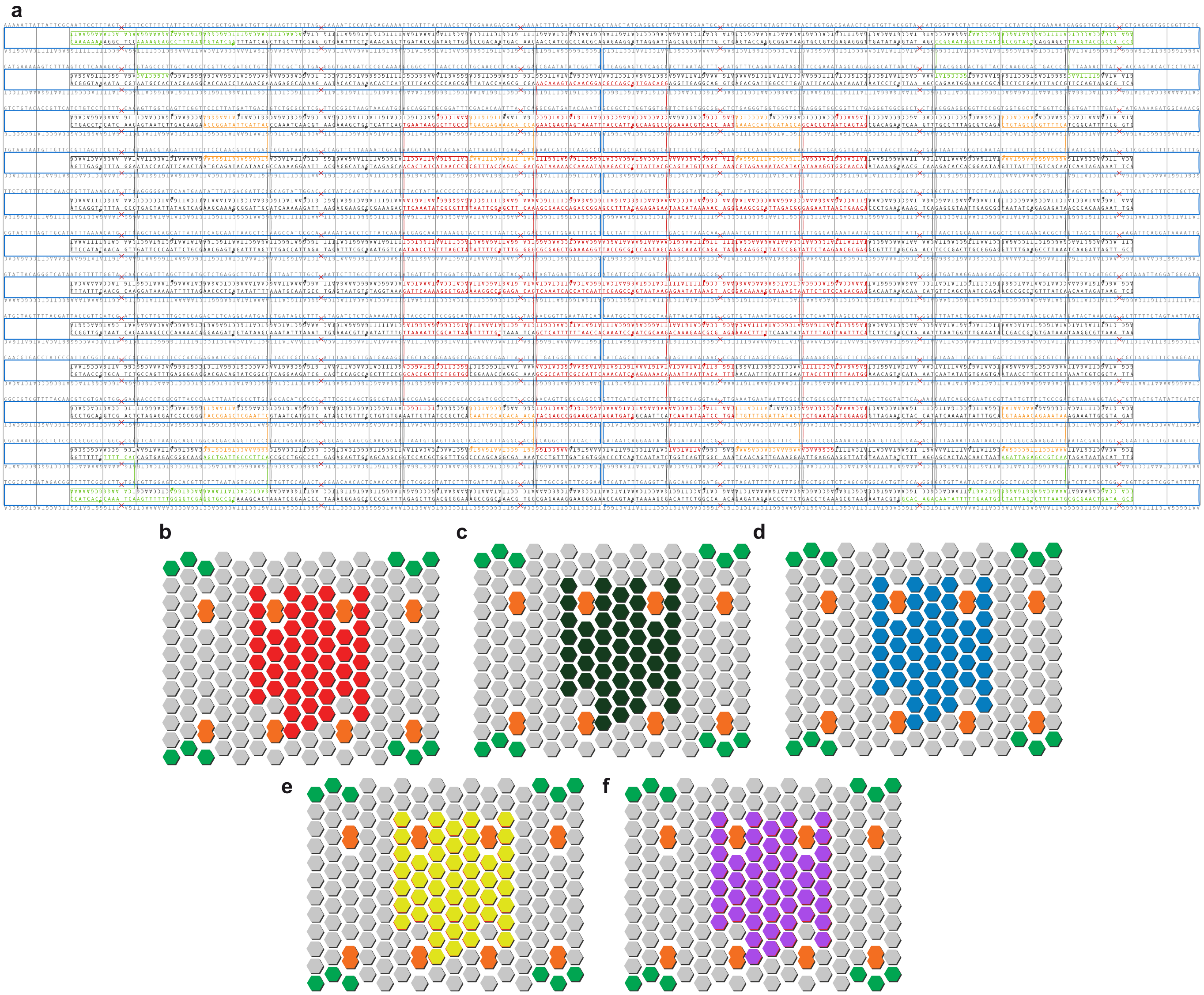

Supplemental Figure 2: Strand diagram and schematics for DNA origami structures. (**a**) Detailed DNA origami strand diagram for *in vitro* mean-shift clustering validation. The origami carries 48 *a* docking strands and 12 fixed Cy3 dyes (hybridized via handle/anti-handle strands). Zoom in to see details. (**b**) Schematic representation for the structure in **a**. Hexagons represent 3' ends of staples. (**c**) DNA origami with 48 *b* docking strands. (**d**) DNA origami with 48 *c* docking strands. (**e**) DNA origami with 48 *d* docking strands. (**f**) DNA origami with 48 *e* docking strands. Strands are colour-coded to denote strand extensions (see **Supplemental Table S1**). Colour code: blue: DNA scaffold; gray: staple strands; red, dark-green, light-blue, yellow and purple: staples with a 3'-handle extension for Exchange-PAINT (docking strands); orange: 5'-biotinylated strands; green: 3'-Cy3-modified staples.

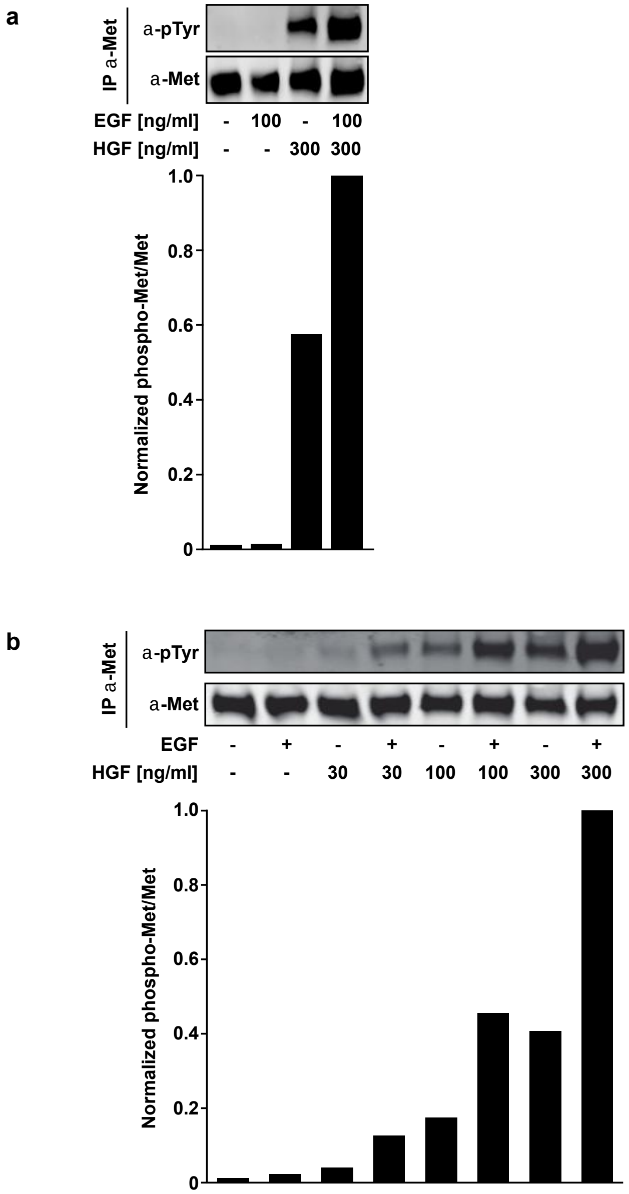

Supplemental Figure 3: Cross-stimulation of Met by EGFR across a range of ligand concentrations. Serum-starved BT20 cells were stimulated, receptor immunoprecipitates were subjected to SDS-PAGE and immunoblots were probed with an anti-phospho-tyrosine antibody or an anti-Met antibody and respective secondary antibodies. Background-subtracted phospho-Met signals were corrected for total Met expression and normalized to the EGF/HGF-treated sample. (**a**) Serum-starved BT20 cells were stimulated for 5 min with 100 ng/ml EGF and/or 300 ng/ml HGF or medium as a control. (**b**) Serum-starved BT20 cells were stimulated for 5 min with 10 µg/ml EGF, different concentrations of HGF, both ligands or medium as a control.

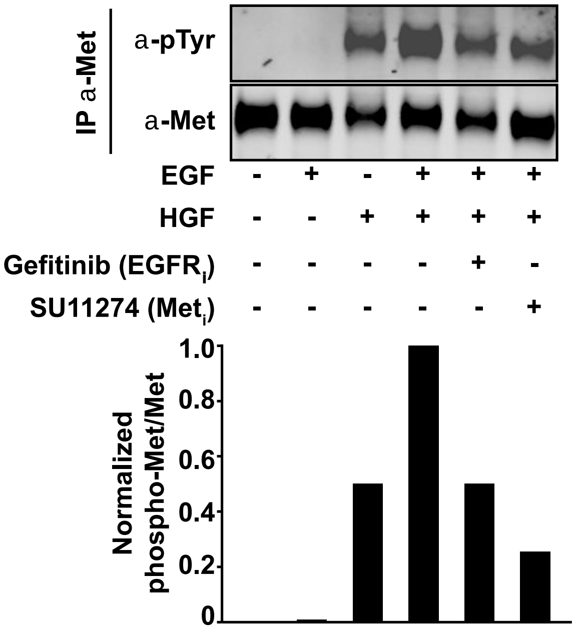

Supplemental Figure 4: Inhibitor experiments in cells co-stimulated with EGF and HGF. Serum-starved BT20 cells were pre-treated for 15 min with 1 µM Gefitinib (EGFR inhibitor) and SU11274 (Met inhibitor) or DMSO as a control and subsequently stimulated for 5 min with 10 µg/ml EGF and/or 300 ng/ml HGF or medium as a control. Receptor immunoprecipitates were subjected to SDS-PAGE and immunoblots were probed with an anti-phospho-tyrosine antibody or an anti-Met antibody and respective secondary antibodies. Background-subtracted phospho-Met signals were corrected for total Met expression and normalized to the EGF/HGF-treated sample.

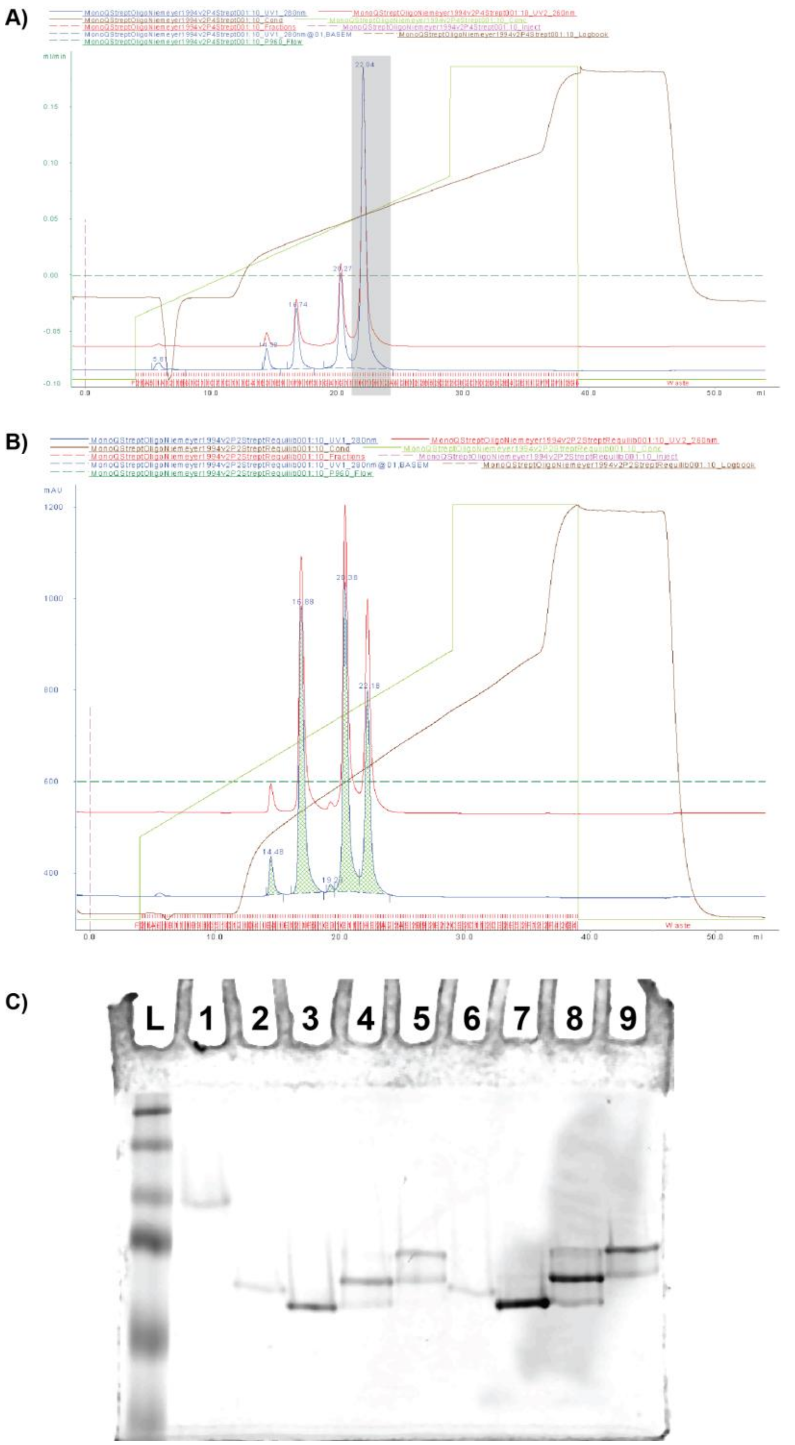

Supplemental Figure 5: Separation of biotinylated oligo-streptavidin conjugates by FPLC and characterization by native gel electrophoresis. (**a**) Initial separation of SAv bound to different numbers of bt-oligos (P1) by MonoQ anion affinity chromatography. The different states elutes at a different volume. SAv (5.81 ml), 1×(bt-oligo)SAv (14.38 ml), 2×(bt-oligo)SAv (16.74 ml), 3×(bt-oligo)SAv (20.27 ml), 4×(bt-oligo)SAv (22.04). (**b**) Re-equilibration of biotinylated oligo-streptavidin conjugates. To increase the fraction of 3×(bt-oligo) conjugates used for coupling to biotinylated antibodies, the 4×(bt-oligo)SAv conjugate peak was collected (grey box in **a**), concentrated and mixed with unlabelled SAv, allowed to equilibrate for 6-12 hours at 37°C and then re-applied to a MonoQ column. The biotinylated oligo from the 4×(bt-oligo)SAv conjugate redistributes to the unlabelled SAv molecules. The appearance of 4 peaks strongly suggests that the fraction used for re-equilibration was the 4×(bt-oligo)SAv state**.** (**c**) Native gel electrophoresis of biotinylated oligo-streptavidin conjugates. Native gel electrophoresis of different bt-oligo-SAv for oligo p1 and p4. Lane 1: unlabelled SAv; lanes 2-5: 1-4×(bt-oligo)SAv for oligo p1; lanes 6-9: 1-4×(bt-oligo)SAv for oligo p4. For up to 2 bound bt-oligos, the added charge results in a higher gel velocity of the bt-oligo-SAv conjugates, but additional bt-oligos increase the gyroscopic radius causing the molecule to run more slowly in the gel. As an example, the 4×(bt-oligo)SAv for oligo p1 has an effective gyroscopic radius of a 180 kDa protein (by size exclusion chromatography, not shown).

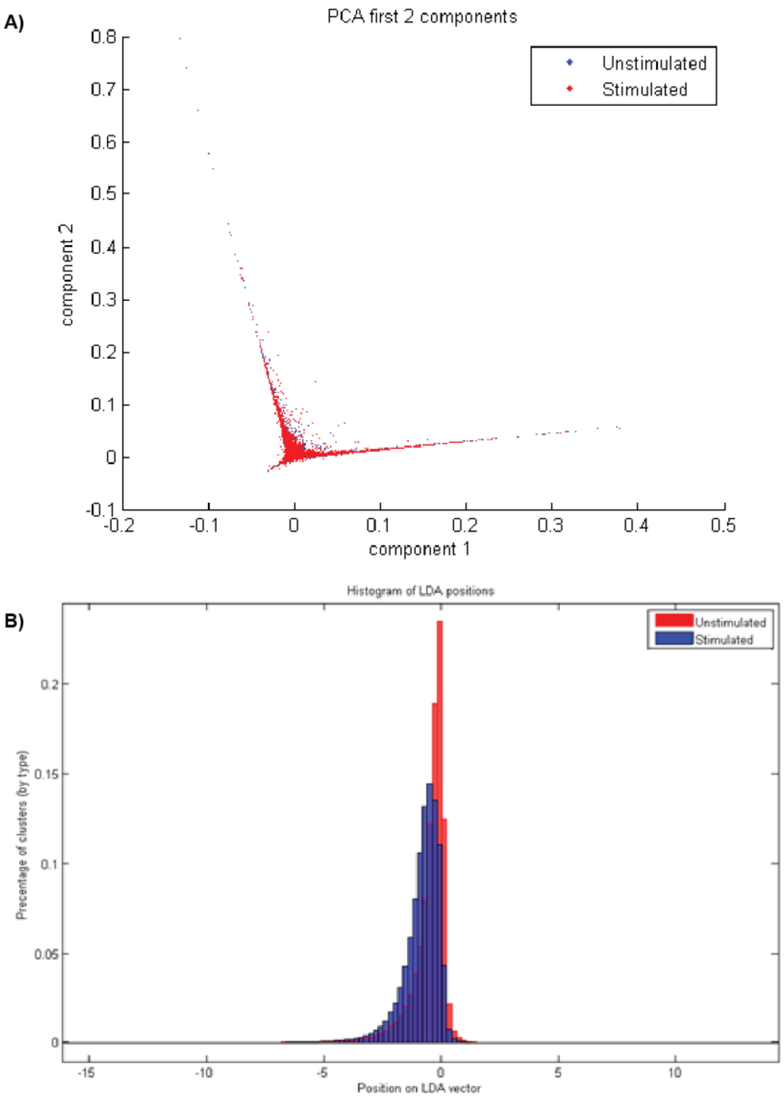

Supplemental Figure 6: Alternative analysis for comparing unstimulated to EGF stimulated receptor clustering data. (**a**) Principle component analysis. Cluster density features for each cluster identified from serum-starved and EGF-stimulated BT20 cells plotted along the first two principle components of the data. (**b**) Linear discriminant analysis. A histogram of position of each cluster identified from serum-starved and EGF-stimulated BT20 cells along the LDA vector. Note that the classes are not well separated.

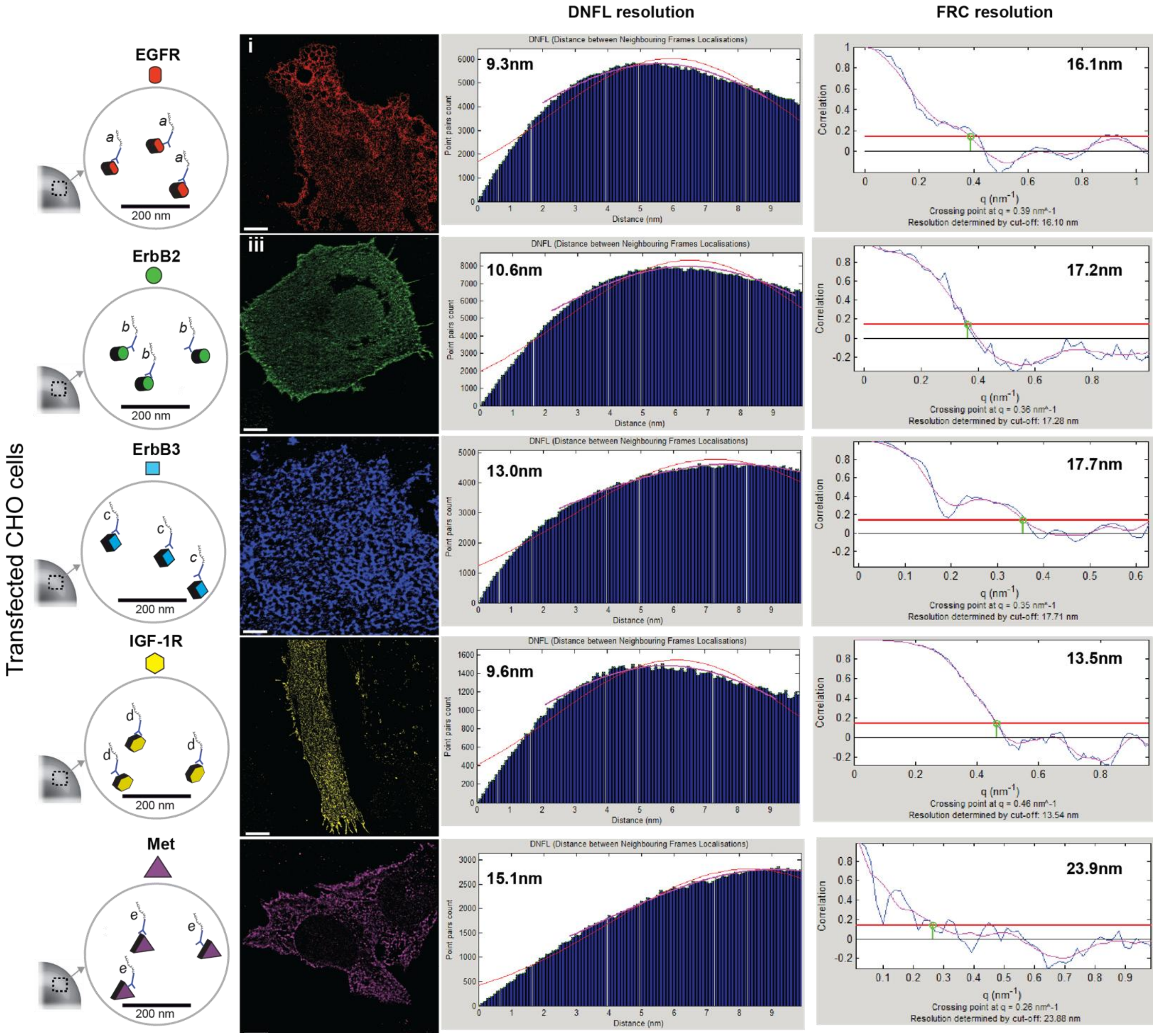

Origami Data

FRC resolution: 19.70 nm **DNFL resolution: 7.5 nm**

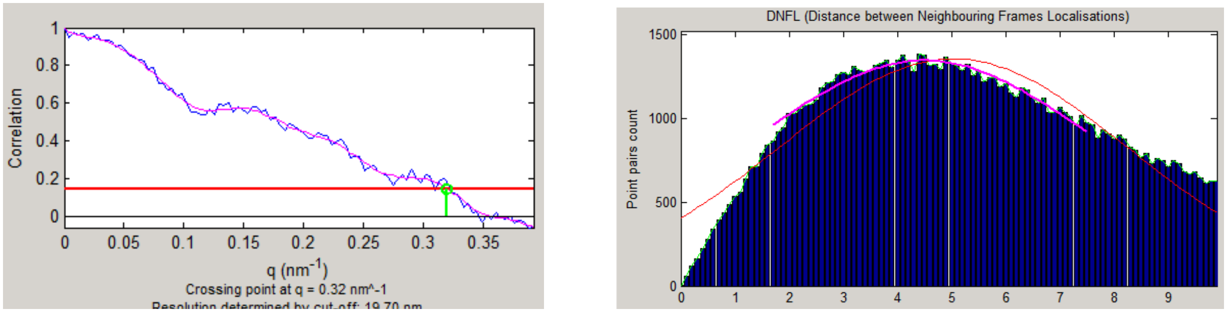

Supplemental Figure 7: Resolution measured by Fourier Ring Correlation and Distance between Neighbouring-Frame Localizations. A comparison of the image resolution for Fig. 1a and Fig. 3b calculated using two different approaches to measuring image resolution FRC and DNFL.

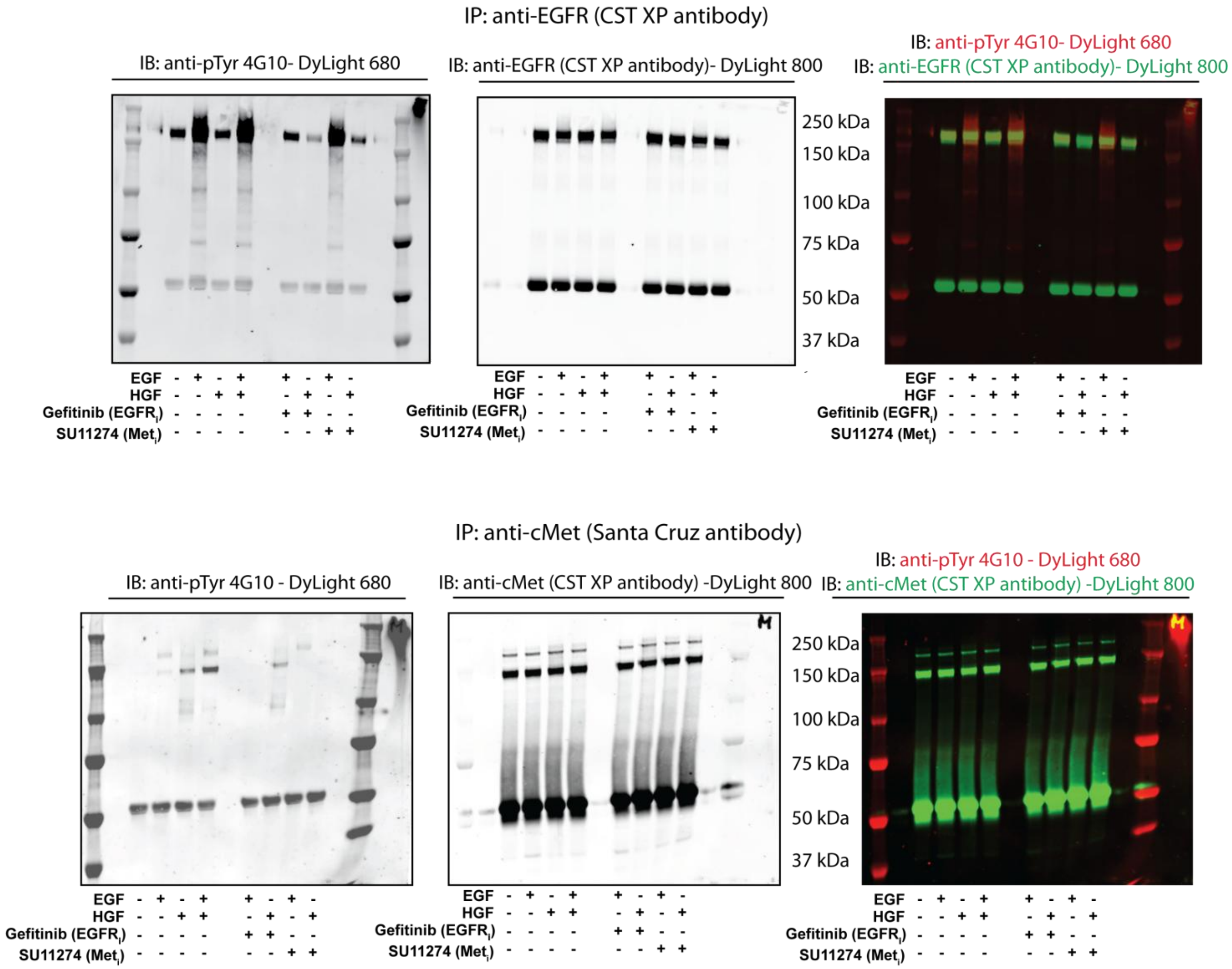

Supplemental Figure 8: Full-length blots from figure 4. Bands (in between ~250 and 150 kDa) from the above blots were presented in Figure 4. Upper panel: anti-EGFR-immunoprecipitation (IP), lower panel: anti-Met-IP. The blots were stained for pTyr with DyLight 680 and the pulled down RTK with DyLight 800. The far right panels show the overlay of the two channels with pTyr in red and RTK in green.

Supplemental Tables

Supplemental Table S1: Staple sequences for 48 docking strands origami with fixed Cy3 dyes. The colour matches the staples in the strand diagram shown in **Supplemental Fig. S2**.

Position	Sequence	Colour	Description
0[111]1[95]	TAAATGAATTTTCTGTATGGGATTAATTTCTT		structure strand
0[143]1[127]	TCTAAAGTTTTGTCGTCTTTCCAGCCGACAA		structure strand
0[175]0[144]	TCCACAGACAGCCCTCATAGTTAGCGTAACGA		structure strand
0[207]1[191]	TCACCAGTACAAACTACAACGCCTAGTACCAG		structure strand
0[239]1[223]	AGGAACCCATGTACCGTAACACTTGATATAA		structure strand
1[32]3[31]	AGGCTCCAGAGGCTTTGAGGACACGGGTAA		structure strand
1[64]4[64]	TTTATCAGGACAGCATCGGAACGACACCAACCTAAAACGAGGTCAATC		structure strand
1[96]3[95]	AAACAGCTTTTTGCGGGATCGTCAACACTAAA		structure strand
1[128]4[128]	TGACAACTCGCTGAGGCTTGCATTATACCAAGCGCGATGATAAA		structure strand
1[160]2[144]	TTAGGATTGGCTGAGACTCCTCAATAACCGAT		structure strand
1[192]4[192]	GCGGATAACCTATTATTCTGAAACAGACGATTGGCCTTGAAGAGCCAC		structure strand
1[224]3[223]	GTATAGCAAACAGTTAATGCCCAATCCTCA		structure strand
1[256]4[256]	CAGGAGGTGGGGTCAGTGCCTTGAGTCTCTGAATTTACCGGGAACCAG		structure strand
2[79]0[80]	CAGCGAAACTTGCTTTCGAGGTGTTGCTAA		structure strand
2[111]0[112]	AAGGCCGCTGATACCGATAGTTGCGACGTTAG		structure strand
2[143]1[159]	ATATTCGGAACCATCGCCCACGCAGAGAAGGA		structure strand
2[175]0[176]	TATTAAGAAGCGGGGTTTTGCTCGTAGCAT		structure strand
2[207]0[208]	TTTCGGAAGTGCCGTCGAGAGGGTGAGTTTCG		structure strand
3[32]5[31]	AATACGTTTGAAAGAGGACAGACTGACCTT		structure strand
3[96]5[95]	ACACTCATCCATGTTACTTAGCCGAAAGCTGC		structure strand
3[224]5[223]	TTAAAGCCAGAGCCGCCACCCTCGACAGAA		structure strand
4[47]2[48]	GACCAACTAATGCCACTACGAAGGGGGTAGCA		structure strand
4[79]2[80]	GCGCAGACAAGAGGCAAAAGAATCCCTCAG		structure strand
4[111]2[112]	GACCTGCTCTTTGACCCCCAGCGAGGGAGTTA		structure strand
4[175]2[176]	CACCAGAAAGGTTGAGGCAGGTCATGAAAG		structure strand
4[207]2[208]	CCACCCTCTATTCACAAACAAATACCTGCCTA		structure strand
4[239]2[240]	GCCTCCCTCAGAATGGAAAGCGCAGTAACAGT		structure strand
4[271]2[272]	AAATCACCTTCCAGTAAGCGTCAGTAATAA		structure strand
5[32]7[31]	CATCAAGTAAAACGAACTAACGAGTTGAGA		structure strand
5[96]7[95]	TCATTCAGATGCGATTTTAAGAACAGGCATAG		structure strand
5[224]7[223]	TCAAGTTTCATTAAAGGTGAATATAAAAGA		structure strand
6[47]4[48]	TACGTTAAAGTAATCTTGACAAGAACCGAACT		structure strand
6[79]4[80]	TTATACCACCAAATCAACGTAACGAACGAG		structure strand
6[239]4[240]	GAAATTATTGCCTTTAGCGTCAGACCGGAACC		structure strand
6[271]4[272]	ACCGATTGTCGGCATTTTCGGTCATAATCA		structure strand
7[32]9[31]	TTTAGGACAAATGCTTTAAACAATCAGGTC		structure strand
7[56]9[63]	ATGCAGATACATAACGGGAATCGTCATAAATAAAGCAAAG		structure strand
7[96]9[95]	TAAGAGCAAATGTTTAGACTGGATAGGAAGCC		structure strand
7[224]9[223]	AACGCAAAGATAGCCGAACAAACCCTGAAC		structure strand
7[248]9[255]	GTTTATTTTGTCACAATCTTACCGAAGCCCTTTAATATCA		structure strand

8[47]6[48]	ATCCCCCTATACCACATTCAACTAGAAAAATC		structure strand
8[79]6[80]	AATACTGCCCAAAAGGAATTACGTGGCTCA		structure strand
8[239]6[240]	AAGTAAGCAGACACCACGGAATAATATTGACG		structure strand
8[271]6[272]	AATAGCTATCAATAGAAAATTCAACATTCA		structure strand
9[32]11[31]	TTTACCCCAACATGTTTTAAATTTCCATAT		structure strand
9[64]11[63]	CGGATTGCAGAGCTTAATTGCTGAAACGAGTA		structure strand
9[96]11[95]	CGAAAGACTTTGATAAGAGGTCATATTTCGCA		structure strand
9[224]11[223]	AAAGTCACAAAATAAACAGCCAGCGTTTTA		structure strand
9[256]11[255]	GAGAGATAGAGCGTCTTTCCAGAGGTTTTGAA		structure strand
10[47]8[48]	CTGTAGCTTGACTATTATAGTCAGTTCATTGA		structure strand
10[79]8[80]	GATGGCTTATCAAAAAGATTAAGAGCGTCC		structure strand
10[239]8[240]	GCCAGTTAGAGGGTAATTGAGCGCTTTAAGAA		structure strand
10[271]8[272]	ACGCTAACACCCACAAGAATTGAAAATAGC		structure strand
11[32]13[31]	AACAGTTTTGTACCAAAAACATTTTATTTC		structure strand
11[64]13[63]	GATTTAGTCAATAAAGCCTCAGAGAACCCTCA		structure strand
11[96]13[95]	AATGGTCAACAGGCAAGGCAAAGAGTAATGTG		structure strand
11[224]13[223]	GCGAACCTCCAAGAACGGGTATGACAATAA		structure strand
11[256]13[255]	GCCTTAAACCAATCAATAATCGGCACGCGCCT		structure strand
12[47]10[48]	TAAATCGGGATTCCCAATTCTGCGATATAATG		structure strand
12[79]10[80]	AAATTAAGTTGACCATTAGATACTTTTGCG		structure strand
12[239]10[240]	CTTATCATTCCCGACTTGCGGGAGCCTAATTT		structure strand
12[271]10[272]	TGTAGAAATCAAGATTAGTTGCTCTTACCA		structure strand
13[32]15[31]	AACGCAAAATCGATGAACGGTACCGGTTGA		structure strand
13[64]15[63]	TATATTTTGTCATTGCCTGAGAGTGGAAGATT		structure strand
13[96]15[95]	TAGGTAAACTATTTTTGAGAGATCAAACGTTA		structure strand
13[224]15[223]	ACAACATGCCAACGCTCAACAGTCTTCTGA		structure strand
13[256]15[255]	GTTTATCAATATGCGTTATACAAACCGACCGT		structure strand
14[47]12[48]	AACAAGAGGGATAAAAATTTTTAGCATAAAGC		structure strand
14[79]12[80]	GCTATCAGAAATGCAATGCCTGAATTAGCA		structure strand
14[239]12[240]	AGTATAAAGTTCAGCTAATGCAGATGTCTTTC		structure strand
14[271]12[272]	TTAGTATCACAATAGATAAGTCCACGAGCA		structure strand
15[32]17[31]	TAATCAGCGGATTGACCGTAATCGTAACCG		structure strand
15[64]18[64]	GTATAAGCCAACCCGTCGGATTCTGACGACAGTATCGGCCGCAAGGCG		structure strand
15[96]17[95]	ATATTTTGGCTTTCATCAACATTATCCAGCCA		structure strand
15[128]18[128]	TAAATCAAAATAATTCGCGTCTCGGAAACCAGGCAAAGGGAAGG		structure strand
15[192]18[192]	TCAAATATAACCTCCGGCTTAGGTAACAATTTCATTTGAAGGCGAATT		structure strand
15[224]17[223]	CCTAAATCAAAATCATAGGTCTAAACAGTA		structure strand
15[256]18[256]	GTGATAAAAAGACGCTGAGAAGAGATAACCTTGCTTCTGTTCGGGAGA		structure strand
16[47]14[48]	ACAAACGGAAAAGCCCCAAAAACACTGGAGCA		structure strand
16[79]14[80]	GCGAGTAAAAATATTTAAATTGTTACAAAG		structure strand
16[239]14[240]	GAATTTATTTAATGGTTTGAAATATTCTTACC		structure strand
16[271]14[272]	CTTAGATTTAAGGCGTTAAATAAAGCCTGT		structure strand
17[32]19[31]	TGCATCTTTCCCAGTCACGACGGCCTGCAG		structure strand
17[96]19[95]	GCTTTCCGATTACGCCAGCTGGCGGCTGTTTC		structure strand
17[224]19[223]	CATAAATCTTTGAATACCAAGTGTTAGAAC		structure strand
18[47]16[48]	CCAGGGTTGCCAGTTTGAGGGGACCCGTGGGA		structure strand

18[79]16[80]	GATGTGCTTCAGGAAGATCGCACAATGTGA		structure strand
18[239]16[240]	CCTGATTGCAATATATGTGAGTGATCAATAGT		structure strand
18[271]16[272]	CTTTTACAAAATCGTCGCTATTAGCGATAG		structure strand
19[32]21[31]	GTCGACTTCGGCCAACGCGCGGGGTTTTTC		structure strand
19[96]21[95]	CTGTGTGATTGCGTTGCGCTCACTAGAGTTGC		structure strand
19[160]20[144]	GCAATTCACATATTCCTGATTATCAAAGTGTA		structure strand
19[224]21[223]	CTACCATAGTTTGAGTAACATTTAAAATAT		structure strand
20[47]18[48]	TTAATGAACTAGAGGATCCCCGGGGGGTAACG		structure strand
20[79]18[80]	TTCCAGTCGTAATCATGGTCATAAAAGGGG		structure strand
20[111]18[112]	CACATTAAAATTGTTATCCGCTCATGCGGGCC		structure strand
20[239]18[240]	ATTTTAAAATCAAAATTATTTGCACGGATTCG		structure strand
20[271]18[272]	CTCGTATTAGAAATTGCGTAGATACAGTAC		structure strand
21[96]23[95]	AGCAAGCGTAGGGTTGAGTGTTGTAGGGAGCC		structure strand
21[120]23[127]	CCCAGCAGGCGAAAAATCCCTTATAAATCAAGCCGGCG		structure strand
21[160]22[144]	TCAATATCGAACCTCAAATATCAATTCCGAAA		structure strand
21[184]23[191]	TCAACAGTTGAAAGGAGCAAATGAAAAATCTAGAGATAGA		structure strand
21[224]23[223]	CTTTAGGGCCTGCAACAGTGCCAATACGTG		structure strand
22[47]20[48]	CTCCAACGCAGTGAGACGGGCAACCAGCTGCA		structure strand
22[79]20[80]	TGGAACAACCGCCTGGCCCTGAGGCCCGCT		structure strand
22[111]20[112]	GCCCGAGAGTCCACGCTGGTTTGCAGCTAACT		structure strand
22[143]21[159]	TCGGCAAATCCTGTTTGATGGTGGACCCTCAA		structure strand
22[175]20[176]	ACCTTGCTTGGTCAGTTGGCAAAGAGCGGA		structure strand
22[207]20[208]	AGCCAGCAATTGAGGAAGGTTATCATCATTTT		structure strand
22[239]20[240]	TTAACACCAGCACTAACAACTAATCGTTATTA		structure strand
22[271]20[272]	CAGAAGATTAGATAATACATTTGTCGACAA		structure strand
23[64]22[80]	AAAGCACTAAATCGGAACCCTAATCCAGTT		structure strand
23[96]22[112]	CCCGATTTAGAGCTTGACGGGGAAAAAGAATA		structure strand
23[128]23[159]	AACGTGGCGAGAAAGGAAGGGAAACCAGTAA		structure strand
23[160]22[176]	TAAAAGGGACATTCTGGCCAACAAAGCATC		structure strand
23[192]22[208]	ACCCTTCTGACCTGAAAGCGTAAGACGCTGAG		structure strand
3[160]4[144]	TTGACAGGCCACCACCAGAGCCGCGATTTGTA		PAINT docking site
4[143]3[159]	TCATCGCCAACAAAGTACAACGGACGCCAGCA		PAINT docking site
5[160]6[144]	GCAAGGCCTCACCAGTAGCACCATGGGCTTGA		PAINT docking site
6[111]4[112]	ATTACCTTTGAATAAGGCTTGCCCAAATCCGC		PAINT docking site
6[143]5[159]	GATGGTTTGAACGAGTAGTAAATTTACCATTA		PAINT docking site
6[175]4[176]	CAGCAAAAGGAAACGTCACCAATGAGCCGC		PAINT docking site
6[207]4[208]	TCACCGACGCACCGTAATCAGTAGCAGAACCG		PAINT docking site
7[120]9[127]	CGTTTACCAGACGACAAAGAAGTTTTGCCATAATTCGA		PAINT docking site
7[160]8[144]	TTATTACGAAGAACTGGCATGATTGCGAGAGG		PAINT docking site
7[184]9[191]	CGTAGAAAATACATACCGAGGAAACGCAATAAGAAGCGCA		PAINT docking site
8[111]6[112]	AATAGTAAACACTATCATAACCCTCATTGTGA		PAINT docking site
8[143]7[159]	CTTTTGCAGATAAAAACCAAAATAAAGACTCC		PAINT docking site
8[175]6[176]	ATACCCAACAGTATGTTAGCAAATTAGAGC		PAINT docking site
8[207]6[208]	AAGGAAACATAAAGGTGGCAACATTATCACCG		PAINT docking site
9[128]11[127]	GCTTCAATCAGGATTAGAGAGTTATTTTCA		PAINT docking site

9[160]10[144]	AGAGAGAAAAAAATGAAAATAGCAAGCAAACT		PAINT docking site
9[192]11[191]	TTAGACGGCCAAATAAGAAACGATAGAAGGCT		PAINT docking site
10[111]8[112]	TTGCTCCTTTCAAATATCGCGTTTGAGGGGGT		PAINT docking site
10[143]9[159]	CCAACAGGAGCGAACCAGACCGGAGCCTTTAC		PAINT docking site
10[175]8[176]	TTAACGTCTAACATAAAAACAGGTAACGGA		PAINT docking site
10[207]8[208]	ATCCCAATGAGAATTAACTGAACAGTTACCAG		PAINT docking site
11[128]13[127]	TTTGGGGATAGTAGTAGCATTAAAAGGCCG		PAINT docking site
11[160]12[144]	CCAATAGCTCATCGTAGGAATCATGGCATCAA		PAINT docking site
11[192]13[191]	TATCCGGTCTCATCGAGAACAAGCGACAAAAG		PAINT docking site
12[111]10[112]	TAAATCATATAACCTGTTTAGCTAACCTTTAA		PAINT docking site
12[143]11[159]	TTCTACTACGCGAGCTGAAAAGGTTACCGCGC		PAINT docking site
12[175]10[176]	TTTTATTTAAGCAAATCAGATATTTTTTGT		PAINT docking site
12[207]10[208]	GTACCGCAATTCTAAGAACGCGAGTATTATTT		PAINT docking site
13[128]15[127]	GAGACAGCTAGCTGATAAATTAATTTTTGT		PAINT docking site
13[160]14[144]	GTAATAAGTTAGGCAGAGGCATTTATGATATT		PAINT docking site
13[192]15[191]	GTAAAGTAATCGCCATATTTAACAAAACTTTT		PAINT docking site
14[111]12[112]	GAGGGTAGGATTCAAAAGGGTGAGACATCCAA		PAINT docking site
14[143]13[159]	CAACCGTTTCAAATCACCATCAATTCGAGCCA		PAINT docking site
14[175]12[176]	CATGTAATAGAATATAAAGTACCAAGCCGT		PAINT docking site
14[207]12[208]	AATTGAGAATTCTGTCCAGACGACTAAACCAA		PAINT docking site
15[160]16[144]	ATCGCAAGTATGTAAATGCTGATGATAGGAAC		PAINT docking site
16[111]14[112]	TGTAGCCATTAAAATTCGCATTAAATGCCGGA		PAINT docking site
16[143]15[159]	GCCATCAAGCTCATTTTTTAACCACAAATCCA		PAINT docking site
16[175]14[176]	TATAACTAACAAAGAACGCGAGAACGCCAA		PAINT docking site
16[207]14[208]	ACCTTTTTATTTTAGTTAATTTCATAGGGCTT		PAINT docking site
17[160]18[144]	AGAAAACAAAGAAGATGATGAAACAGGCTGCG		PAINT docking site
18[111]16[112]	TCTTCGCTGCACCGCTTCTGGTGCGGCCTTCC		PAINT docking site
18[143]17[159]	CAACTGTTGCGCCATTCGCCATTCAAACATCA		PAINT docking site
18[175]16[176]	CTGAGCAAAAATTAATTACATTTTGGGTTA		PAINT docking site
18[207]16[208]	CGCGCAGATTACCTTTTTTAATGGGAGAGACT		PAINT docking site
20[143]19[159]	AAGCCTGGTACGAGCCGGAAGCATAGATGATG		PAINT docking site
20[175]18[176]	ATTATCATTCAATATAATCCTGACAATTAC		PAINT docking site
20[207]18[208]	GCGGAACATCTGAATAATGGAAGGTACAAAAT		PAINT docking site
0[47]1[31]	AGAAAGGAACAACTAAAGGAATTCAAAAAAA		3’-Cy3 modification
0[79]1[63]	ACAACTTTCAACAGTTTCAGCGGATGTATCGG		3’-Cy3 modification
0[271]1[255]	CCACCCTCATTTTCAGGGATAGCAACCGTACT		3’-Cy3 modification
2[47]0[48]	ACGGCTACAAAAGGAGCCTTTAATGTGAGAAT		3’-Cy3 modification
2[239]0[240]	GCCCGTATCCGGAATAGGTGTATCAGCCCAAT		3’-Cy3 modification
2[271]0[272]	GTTTTAACTTAGTACCGCCACCCAGAGCCA		3’-Cy3 modification
21[32]23[31]	TTTTCACTCAAAGGGCGAAAAACCATCACC		3’-Cy3 modification
21[56]23[63]	AGCTGATTGCCCTTCAGAGTCCACTATTAAAGGGTGCCGT		3’-Cy3 modification
21[248]23[255]	AGATTAGAGCCGTCAAAAAACAGAGGTGAGGCCTATTAGT		3’-Cy3 modification
23[32]22[48]	CAAATCAAGTTTTTTGGGGTCGAAACGTGGA		3’-Cy3 modification
23[224]22[240]	GCACAGACAATATTTTTGAATGGGGTCAGTA		3’-Cy3 modification
23[256]22[272]	CTTTAATGCGCGAACTGATAGCCCCACCAG		3’-Cy3 modification

4[63]6[56]	ATAAGGGAACCGGATATTCATTACGTCAGGACGTTGGGAA		5’-Biotin modification
4[127]6[120]	TTGTGTCGTGACGAGAAACACCAAATTTCAACTTTAAT		5’-Biotin modification
4[191]6[184]	CACCCTCAGAAACCATCGATAGCATTGAGCCATTTGGGAA		5’-Biotin modification
4[255]6[248]	AGCCACCACTGTAGCGCGTTTTCAAGGGAGGGAAGGTAAA		5’-Biotin modification
18[63]20[56]	ATTAAGTTTACCGAGCTCGAATTCGGGAAACCTGTCGTGC		5’-Biotin modification
18[127]20[120]	GCGATCGGCAATTCCACACAACAGGTGCCTAATGAGTG		5’-Biotin modification
18[191]20[184]	ATTCATTTTTGTTTGGATTATACTAAGAAACCACCAGAAG		5’-Biotin modification
18[255]20[248]	AACAATAACGTAAAACAGAAATAAAAATCCTTTGCCCGAA		5’-Biotin modification

Supplemental Table S2: M13mp18 scaffold sequence for DNA origami structures.

TTCCCTTCCTTTCTCGCCACGTTCGCCGGCTTTCCCCGTCAAGCTCTAAATCGGGGGCTCCCTTTAGGGTTCCGATTTAGTGCTTTACGGCACCTCGACCCCAAAAAAC
TTGATTTGGGTGATGGTTCACGTAGTGGGCCATCGCCCTGATAGACGGTTTTTCGCCCTTTGACGTTGGAGTCCACGTTCTTTAATAGTGGACTCTTGTTCCAAACTGG
AACAACACTCAACCCTATCTCGGGCTATTCTTTTGATTTATAAGGGATTTTGCCGATTTCGGAACCACCATCAAACAGGATTTTCGCCTGCTGGGGCAAACCAGCGTGG
ACCGCTTGCTGCAACTCTCTCAGGGCCAGGCGGTGAAGGGCAATCAGCTGTTGCCCGTCTCACTGGTGAAAAGAAAAACCACCCTGGCGCCCAATACGCAAACCGCCTC
TCCCCGCGCGTTGGCCGATTCATTAATGCAGCTGGCACGACAGGTTTCCCGACTGGAAAGCGGGCAGTGAGCGCAACGCAATTAATGTGAGTTAGCTCACTCATTAGGC
ACCCCAGGCTTTACACTTTATGCTTCCGGCTCGTATGTTGTGTGGAATTGTGAGCGGATAACAATTTCACACAGGAAACAGCTATGACCATGATTACGAATTCGAGCTC
GGTACCCGGGGATCCTCTAGAGTCGACCTGCAGGCATGCAAGCTTGGCACTGGCCGTCGTTTTACAACGTCGTGACTGGGAAAACCCTGGCGTTACCCAACTTAATCGC
CTTGCAGCACATCCCCCTTTCGCCAGCTGGCGTAATAGCGAAGAGGCCCGCACCGATCGCCCTTCCCAACAGTTGCGCAGCCTGAATGGCGAATGGCGCTTTGCCTGGT
TTCCGGCACCAGAAGCGGTGCCGGAAAGCTGGCTGGAGTGCGATCTTCCTGAGGCCGATACTGTCGTCGTCCCCTCAAACTGGCAGATGCACGGTTACGATGCGCCCAT
CTACACCAACGTGACCTATCCCATTACGGTCAATCCGCCGTTTGTTCCCACGGAGAATCCGACGGGTTGTTACTCGCTCACATTTAATGTTGATGAAAGCTGGCTACAG
GAAGGCCAGACGCGAATTATTTTTGATGGCGTTCCTATTGGTTAAAAAATGAGCTGATTTAACAAAAATTTAATGCGAATTTTAACAAAATATTAACGTTTACAATTTA
AATATTTGCTTATACAATCTTCCTGTTTTTGGGGCTTTTCTGATTATCAACCGGGGTACATATGATTGACATGCTAGTTTTACGATTACCGTTCATCGATTCTCTTGTT
TGCTCCAGACTCTCAGGCAATGACCTGATAGCCTTTGTAGATCTCTCAAAAATAGCTACCCTCTCCGGCATTAATTTATCAGCTAGAACGGTTGAATATCATATTGATG
GTGATTTGACTGTCTCCGGCCTTTCTCACCCTTTTGAATCTTTACCTACACATTACTCAGGCATTGCATTTAAAATATATGAGGGTTCTAAAAATTTTTATCCTTGCGT
TGAAATAAAGGCTTCTCCCGCAAAAGTATTACAGGGTCATAATGTTTTTGGTACAACCGATTTAGCTTTATGCTCTGAGGCTTTATTGCTTAATTTTGCTAATTCTTTG
CCTTGCCTGTATGATTTATTGGATGTTAATGCTACTACTATTAGTAGAATTGATGCCACCTTTTCAGCTCGCGCCCCAAATGAAAATATAGCTAAACAGGTTATTGACC
ATTTGCGAAATGTATCTAATGGTCAAACTAAATCTACTCGTTCGCAGAATTGGGAATCAACTGTTATATGGAATGAAACTTCCAGACACCGTACTTTAGTTGCATATTT
AAAACATGTTGAGCTACAGCATTATATTCAGCAATTAAGCTCTAAGCCATCCGCAAAAATGACCTCTTATCAAAAGGAGCAATTAAAGGTACTCTCTAATCCTGACCTG
TTGGAGTTTGCTTCCGGTCTGGTTCGCTTTGAAGCTCGAATTAAAACGCGATATTTGAAGTCTTTCGGGCTTCCTCTTAATCTTTTTGATGCAATCCGCTTTGCTTCTG
ACTATAATAGTCAGGGTAAAGACCTGATTTTTGATTTATGGTCATTCTCGTTTTCTGAACTGTTTAAAGCATTTGAGGGGGATTCAATGAATATTTATGACGATTCCGC
AGTATTGGACGCTATCCAGTCTAAACATTTTACTATTACCCCCTCTGGCAAAACTTCTTTTGCAAAAGCCTCTCGCTATTTTGGTTTTTATCGTCGTCTGGTAAACGAG
GGTTATGATAGTGTTGCTCTTACTATGCCTCGTAATTCCTTTTGGCGTTATGTATCTGCATTAGTTGAATGTGGTATTCCTAAATCTCAACTGATGAATCTTTCTACCT
GTAATAATGTTGTTCCGTTAGTTCGTTTTATTAACGTAGATTTTTCTTCCCAACGTCCTGACTGGTATAATGAGCCAGTTCTTAAAATCGCATAAGGTAATTCACAATG
ATTAAAGTTGAAATTAAACCATCTCAAGCCCAATTTACTACTCGTTCTGGTGTTTCTCGTCAGGGCAAGCCTTATTCACTGAATGAGCAGCTTTGTTACGTTGATTTGG
GTAATGAATATCCGGTTCTTGTCAAGATTACTCTTGATGAAGGTCAGCCAGCCTATGCGCCTGGTCTGTACACCGTTCATCTGTCCTCTTTCAAAGTTGGTCAGTTCGG
TTCCCTTATGATTGACCGTCTGCGCCTCGTTCCGGCTAAGTAACATGGAGCAGGTCGCGGATTTCGACACAATTTATCAGGCGATGATACAAATCTCCGTTGTACTTTG
TTTCGCGCTTGGTATAATCGCTGGGGGTCAAAGATGAGTGTTTTAGTGTATTCTTTTGCCTCTTTCGTTTTAGGTTGGTGCCTTCGTAGTGGCATTACGTATTTTACCC
GTTTAATGGAAACTTCCTCATGAAAAAGTCTTTAGTCCTCAAAGCCTCTGTAGCCGTTGCTACCCTCGTTCCGATGCTGTCTTTCGCTGCTGAGGGTGACGATCCCGCA
AAAGCGGCCTTTAACTCCCTGCAAGCCTCAGCGACCGAATATATCGGTTATGCGTGGGCGATGGTTGTTGTCATTGTCGGCGCAACTATCGGTATCAAGCTGTTTAAGA
AATTCACCTCGAAAGCAAGCTGATAAACCGATACAATTAAAGGCTCCTTTTGGAGCCTTTTTTTTGGAGATTTTCAACGTGAAAAAATTATTATTCGCAATTCCTTTAG
TTGTTCCTTTCTATTCTCACTCCGCTGAAACTGTTGAAAGTTGTTTAGCAAAATCCCATACAGAAAATTCATTTACTAACGTCTGGAAAGACGACAAAACTTTAGATCG
TTACGCTAACTATGAGGGCTGTCTGTGGAATGCTACAGGCGTTGTAGTTTGTACTGGTGACGAAACTCAGTGTTACGGTACATGGGTTCCTATTGGGCTTGCTATCCCT
GAAAATGAGGGTGGTGGCTCTGAGGGTGGCGGTTCTGAGGGTGGCGGTTCTGAGGGTGGCGGTACTAAACCTCCTGAGTACGGTGATACACCTATTCCGGGCTATACTT
ATATCAACCCTCTCGACGGCACTTATCCGCCTGGTACTGAGCAAAACCCCGCTAATCCTAATCCTTCTCTTGAGGAGTCTCAGCCTCTTAATACTTTCATGTTTCAGAA
TAATAGGTTCCGAAATAGGCAGGGGGCATTAACTGTTTATACGGGCACTGTTACTCAAGGCACTGACCCCGTTAAAACTTATTACCAGTACACTCCTGTATCATCAAAA
GCCATGTATGACGCTTACTGGAACGGTAAATTCAGAGACTGCGCTTTCCATTCTGGCTTTAATGAGGATTTATTTGTTTGTGAATATCAAGGCCAATCGTCTGACCTGC
CTCAACCTCCTGTCAATGCTGGCGGCGGCTCTGGTGGTGGTTCTGGTGGCGGCTCTGAGGGTGGTGGCTCTGAGGGTGGCGGTTCTGAGGGTGGCGGCTCTGAGGGAGG
CGGTTCCGGTGGTGGCTCTGGTTCCGGTGATTTTGATTATGAAAAGATGGCAAACGCTAATAAGGGGGCTATGACCGAAAATGCCGATGAAAACGCGCTACAGTCTGAC
GCTAAAGGCAAACTTGATTCTGTCGCTACTGATTACGGTGCTGCTATCGATGGTTTCATTGGTGACGTTTCCGGCCTTGCTAATGGTAATGGTGCTACTGGTGATTTTG
CTGGCTCTAATTCCCAAATGGCTCAAGTCGGTGACGGTGATAATTCACCTTTAATGAATAATTTCCGTCAATATTTACCTTCCCTCCCTCAATCGGTTGAATGTCGCCC
TTTTGTCTTTGGCGCTGGTAAACCATATGAATTTTCTATTGATTGTGACAAAATAAACTTATTCCGTGGTGTCTTTGCGTTTCTTTTATATGTTGCCACCTTTATGTAT
GTATTTTCTACGTTTGCTAACATACTGCGTAATAAGGAGTCTTAATCATGCCAGTTCTTTTGGGTATTCCGTTATTATTGCGTTTCCTCGGTTTCCTTCTGGTAACTTT
GTTCGGCTATCTGCTTACTTTTCTTAAAAAGGGCTTCGGTAAGATAGCTATTGCTATTTCATTGTTTCTTGCTCTTATTATTGGGCTTAACTCAATTCTTGTGGGTTAT
CTCTCTGATATTAGCGCTCAATTACCCTCTGACTTTGTTCAGGGTGTTCAGTTAATTCTCCCGTCTAATGCGCTTCCCTGTTTTTATGTTATTCTCTCTGTAAAGGCTG
CTATTTTCATTTTTGACGTTAAACAAAAAATCGTTTCTTATTTGGATTGGGATAAATAATATGGCTGTTTATTTTGTAACTGGCAAATTAGGCTCTGGAAAGACGCTCG
TTAGCGTTGGTAAGATTCAGGATAAAATTGTAGCTGGGTGCAAAATAGCAACTAATCTTGATTTAAGGCTTCAAAACCTCCCGCAAGTCGGGAGGTTCGCTAAAACGCC
TCGCGTTCTTAGAATACCGGATAAGCCTTCTATATCTGATTTGCTTGCTATTGGGCGCGGTAATGATTCCTACGATGAAAATAAAAACGGCTTGCTTGTTCTCGATGAG
TGCGGTACTTGGTTTAATACCCGTTCTTGGAATGATAAGGAAAGACAGCCGATTATTGATTGGTTTCTACATGCTCGTAAATTAGGATGGGATATTATTTTTCTTGTTC
AGGACTTATCTATTGTTGATAAACAGGCGCGTTCTGCATTAGCTGAACATGTTGTTTATTGTCGTCGTCTGGACAGAATTACTTTACCTTTTGTCGGTACTTTATATTC
TCTTATTACTGGCTCGAAAATGCCTCTGCCTAAATTACATGTTGGCGTTGTTAAATATGGCGATTCTCAATTAAGCCCTACTGTTGAGCGTTGGCTTTATACTGGTAAG
AATTTGTATAACGCATATGATACTAAACAGGCTTTTTCTAGTAATTATGATTCCGGTGTTTATTCTTATTTAACGCCTTATTTATCACACGGTCGGTATTTCAAACCAT
TAAATTTAGGTCAGAAGATGAAATTAACTAAAATATATTTGAAAAAGTTTTCTCGCGTTCTTTGTCTTGCGATTGGATTTGCATCAGCATTTACATATAGTTATATAAC
CCAACCTAAGCCGGAGGTTAAAAAGGTAGTCTCTCAGACCTATGATTTTGATAAATTCACTATTGACTCTTCTCAGCGTCTTAATCTAAGCTATCGCTATGTTTTCAAG
GATTCTAAGGGAAAATTAATTAATAGCGACGATTTACAGAAGCAAGGTTATTCACTCACATATATTGATTTATGTACTGTTTCCATTAAAAAAGGTAATTCAAATGAAA
TTGTTAAATGTAATTAATTTTGTTTTCTTGATGTTTGTTTCATCATCTTCTTTTGCTCAGGTAATTGAAATGAATAATTCGCCTCTGCGCGATTTTGTAACTTGGTATT
CAAAGCAATCAGGCGAATCCGTTATTGTTTCTCCCGATGTAAAAGGTACTGTTACTGTATATTCATCTGACGTTAAACCTGAAAATCTACGCAATTTCTTTATTTCTGT
TTTACGTGCAAATAATTTTGATATGGTAGGTTCTAACCCTTCCATTATTCAGAAGTATAATCCAAACAATCAGGATTATATTGATGAATTGCCATCATCTGATAATCAG
GAATATGATGATAATTCCGCTCCTTCTGGTGGTTTCTTTGTTCCGCAAAATGATAATGTTACTCAAACTTTTAAAATTAATAACGTTCGGGCAAAGGATTTAATACGAG

TTGTCGAATTGTTTGTAAAGTCTAATACTTCTAAATCCTCAAATGTATTATCTATTGACGGCTCTAATCTATTAGTTGTTAGTGCTCCTAAAGATATTTTAGATAACCT
TCCTCAATTCCTTTCAACTGTTGATTTGCCAACTGACCAGATATTGATTGAGGGTTTGATATTTGAGGTTCAGCAAGGTGATGCTTTAGATTTTTCATTTGCTGCTGGC
TCTCAGCGTGGCACTGTTGCAGGCGGTGTTAATACTGACCGCCTCACCTCTGTTTTATCTTCTGCTGGTGGTTCGTTCGGTATTTTTAATGGCGATGTTTTAGGGCTAT
CAGTTCGCGCATTAAAGACTAATAGCCATTCAAAAATATTGTCTGTGCCACGTATTCTTACGCTTTCAGGTCAGAAGGGTTCTATCTCTGTTGGCCAGAATGTCCCTTT
TATTACTGGTCGTGTGACTGGTGAATCTGCCAATGTAAATAATCCATTTCAGACGATTGAGCGTCAAAATGTAGGTATTTCCATGAGCGTTTTTCCTGTTGCAATGGCT
GGCGGTAATATTGTTCTGGATATTACCAGCAAGGCCGATAGTTTG

Supplemental Table S3: DNA-PAINT docking and imager sequences and biotin docking sequence.

Description	Sequence
Imager a*	5′-CTAGATGTAT-dye
Imager b*	5′-GTAGATTCAT-dye
Imager c*	5'-CATACATTGA-dye
Imager d*	5'-CACCTTATTA-dye
Imager e*	5'-CCTTCTCTAT-dye
Biotinylated a docking site for antibody EGFR coupling (a)	Biotin-TTATACATCTA-3′
Biotinylated b docking site for antibody ErbB2 coupling (b)	Biotin-TTATGAATCTA-3′
Biotinylated c docking site for antibody ErbB3 coupling (c)	Biotin-TTTCAATGTAT-3′
Biotinylated d docking site for antibody IGF-1R coupling (d)	Biotin-TTTAATAAGGT-3′
Biotinylated e docking site for antibody Met coupling (e)	Biotin-TTATAGAGAAG-3′
9nt a docking site	Strand-TTATACATCTA-3′
9nt b docking site	Strand-TTATGAATCTA-3′
9nt c docking site	Strand-TTTCAATGTAT-3′
9nt d docking site	Strand-TTTAATAAGGT-3′
9nt e docking site	Strand-TTATAGAGAAG-3′
Handle strand for binding of “fixed” Cy3-labelled strand	Staple-TAACATTCCTAACTTCTCATA
Cy3-labelled anti-handle strand	5′-TATGAGAAGTTAGGAATGTTA-Cy3

Supplemental Protocol 1: Antibody-DNA conjugates.

General Scheme and principles:
In order to generate specific antibody-DNA conjugates, we used a streptavidin (SAv) bridge to couple a biotinylated antibody (bt-Ab) and a biotin-conjugated oligo (bt-oligo). However, when SAv is directly mixed with bt-Ab (even at 10:1 ratio of SAv:bt-Ab) the antibodies will often cross-link with other antibodies because SAv is a tetramer allowing it to form large and undesirable precipitates. Here we provide the detailed process by which in a first step, SAv was coupled to exactly 3 bt-oligos leaving only one free biotin binding site for the antibody. This ensures that antibody aggregates will not form. We then describe the process of coupling this 3×(bt-oligo)/SAv conjugate to the bt-Ab and preparing it for long-term storage.

Preparation and separation of 3×(bt-oligo)/SAv:
SAv was mixed with bt-oligos at a 1:3 molar ratio for 30 min at room temperature. This should ideally maximize the amount of the SAv in the 3×(bt-oligo)SAv state, approximately 40% estimated by the binomial distribution.

Separation of biotinylated oligo-streptavidin conjugates by MonoQ anion affinity chromatography:
Based on Niemeyer *et. al.*[1], the following buffers were prepared: buffer A: 20 mM Tris pH 6.3, 0.3 M NaCl; buffer B: 20 mM Tris pH 6.3, 1.0 M NaCl. A MonoQ 5/50 column was washed with 1 ml of 2M NaCl and then equilibrated with 15 ml of Buffer A. The bt-oligo-SAv reaction was brought to 20 mM Tris pH 6.3, diluted in buffer A to 400 µl and injected onto the 1 ml injection loop of a ÄKTA Explorer (GE Healthcare). The loop was placed into the flow path, the flow rate was set to 0.2 ml/min, 5 ml of buffer A were applied, then Buffer B was added up to a 20% concentration, followed by a linear gradient between 20% and 40-65% of buffer B over the course of 25 ml while collecting 200 µl fractions into 96 well plates with 10 µl of 1 M Tris pH 7.5. Finally, the concentration of buffer B was jumped to 100% for 10 ml and then the column was equilibrated with 15 ml of buffer A (at 2.0 ml/min). As seen in **Supplemental Fig. S5a**, unlabelled SAv does not bind to the column (volume 5.6 ml) and the following four peaks elute with increasing NaCl concentration representing the 1×, 2×, 3× and 4×(bt-oligo)SAv conjugates. This result was confirmed by SAv re-equilibration (described below, see **Supplemental Fig. S5b**) and by native gel electrophoresis (**Supplemental Fig. S5c)**. The fractions collected should be approximately at pH 7.4 with 70 mM Tris. The 3×(bt-oligo)SAv conjugate peak was saved for antibody coupling. To improve the yield, the 4×(bt-oligo)SAV conjugate peak was used for re-equilibration (described below).

Native Electrophoresis:
Gels were cast as a 10% running gel at pH 8.8 and a 4.5% stacking gel at pH 6.8 in Tris buffer. Samples were diluted in 4× loading buffer (4× Tris buffer pH 6.8, 40% glycerol, 10% saturated bromophenol blue solution). The gel running buffer was a standard Tris-Glycine buffer without SDS. See results in **Supplemental Fig. S5c**.

SAv re-equilibration:
As seen in **Supplemental Fig. S5a**, a significant amount of bt-oligos was lost in the 4×(bt-oligo)SAv conjugate peak. We could recover these conjugates by a re-equilibration process. This was demonstrated in Jones *et. al.*[2] where 4×bt-SAv was mixed with unlabelled SAv and incubated at room temperature for 3 days. After this incubation time, the bound form of SAv reached a steady state that is equivalent to mixing the SAv tetramer with biotin at a 1:2 molar ratio. As shown in **Supplemental Fig. S5b,** the 4×(bt-oligo)SAv conjugate peak was collected, concentrated and mixed with unlabelled SAv to an approximate 1:3 ratio of SAv to 4×(bt-oligo)SAv and allowed to equilibrate at 37 °C for 6-12 hours. Then the mixture was brought approximately to pH6.3 and diluted with dH_20 and reapplied to the MonoQ column. The 3×(bt-oligo)SAv conjugate peak was collected and combined with the saved 3×(bt-oligo)SAv conjugate from the first MonoQ separation step.

Coupling 3×(bt-oligo)SAv conjugates to biotinylated antibodies:
As described above, bt-oligos were mixed with SAv and fractionated by MonoQ columns and re-equilibrated if necessary. The 3×(bt-oligo)SAv conjugate fractions were collected and the approximate quantity was determined. This was done making two assumptions: (1) SAv recovery is ~ 90% and (2) the area under the curve for absorbance at 280 nm (Abs280) is proportional to the SAv concentration. Then the fraction of 3×(bt-oligo)SAv conjugates equals AreaAbs280(3×(bt-oligo)SAv)/AreaAbs280(total bt-oligo-SAv). Note that this a crude estimate of the SAv quantity because the bt-oligos have a significant absorbance at 280 nm. The concentration of 3×(bt-oligo)SAv conjugates was used to determine how much bt-Ab should be added to generate a 1:2 antibody to 3×(bt-oligo)SAv molar ratio. The antibody was added to the 3×(bt-oligo)SAv fractions and incubated at room temperature by rotation end over end for

30 min. The antibody-DNA conjugate was then concentrated using 4 ml Amicon spin concentrators (Millipore) with a 10 kDa molecular weight cut-off for 15-20 min until the concentration of the antibody-DNA conjugate was > 1.0 mg/ml. BSA and Na-Azide were added to a concentration of 2mg/ml and 0.4%, respectively and the antibody-DNA conjugate was brought to a concentration of 1 mg of antibody/ml. This solution was then mixed in a 1:1 ratio with glycerol by gently pipetting, yielding a final concentration of 0.5 mg/ml in ~35 mM Tris pH 7.4, 300 mM NaCl, 1 mg/ml BSA, 0.2% Na-Azide and 50% Glycerol. These antibody-DNA conjugate stocks were aliquoted and stored at -20 °C.

Supplemental Protocol 2: Measuring receptor clustering distortion during Exchange-PAINT imaging.

Distortion of apparent receptor clusters was measured comparing two rounds of imaging of EGFR in the same BT20 cell, once at the beginning and again after 5 rounds of Exchange-PAINT imaging. This allows for the quantification of possible clustering distortion after several washing steps (**Supplemental Fig. S1b-c**). Imaging was performed at 2.5nM using Atto655-labelled imager strands, 7,500 frames per cycle, 10 Hz imaging rate and washing of 1-2 minutes per cycle.

Sample distortion and receptor sampling were assessed using image cross-correlation. A normalized cross-correlation coefficient was calculated between the images obtained in the first and last round. The same region of interest (ROI), highlighted by a red rectangle in **Supplemental Fig. S1b** and **S1c** was selected in each of the two images. The cross-correlation coefficient was determined to be 0.8348. As described previously[3], even without liquid exchange, one cannot expect 100 % correlation between two consecutive images due to the stochastic nature of image acquisition.

Supplemental Note: Rationale for choosing Random Forests analysis.

We attempted several methods for analyzing and interpreting the difference between the receptor clusters found in unstimulated and EGF-stimulated BT20 cells. The other approaches that we took fell into two general categories, dimensionality reduction and unsupervised learning. The first category comprised techniques like principle component analysis (PCA) and linear discriminant analysis (LDA) that finds the vectors that have the largest variance and best separate the two classes, respectively.

The results of the PCA were difficult to interpret due to the lack of distinction between the two classes of unstimulated and EGF-stimulated cells and no obvious separation when the data is plotted along the first two components (**Supplemental Fig. S6a**). Also worth noting is that the first five components were dominated by a single feature.

LDA returns a high dimensional vector that best separates the two classes. The separator between the two classes the largest components by magnitude are d(IGF-1R)*d(Met), d(EGFR)*d(Met), d(ErbB3)*d(Met). We chose not to use this analysis because it was not able separate the two classes of unstimulated and EGF-stimulated cells (**Supplemental Fig. S6b**) and interpreting the magnitudes of the LDA was difficult because each feature had different ranges. We repeated analysis after normalizing each feature so that they all had a maximum of 1 and a minimum of 0 and found that the most important separating features were d(IGF-1R)*d(IGF-1R), d(Met)*d(Met) and d(IGF-1R).

We also investigated using unsupervised learning techniques, which generally cluster data into distinct groups in feature space. We tried two techniques: mean-shift clustering and unsupervised learning of Gaussian mixture models[4]. The mean-shift clustering always yielded 1 cluster with over 99.9%, thereby failed to make meaningful distinctions. Mixture modelling yielded a set of overlapping clusters and was difficult to interpret.

The difficulty of using and interpreting the results of these techniques likely stems from the nature of the data. As mentioned in the main text the data is highly heterogeneous and we had little expectation of the appearance of a distinct cluster type after stimulation and even If a distinctive cluster type did appear we expected that it would represent a small part of the overall population of clusters. The former property causes problems for the unsupervised learning approaches and the latter property is a challenge for the dimensionality reduction approaches. We chose Random Forests analysis because it is well suited to handle heterogeneous data and is relatively intuitive to interpret.

Supplemental Note: Dissociation rate of streptavidin-biotin bond.

The off rate (k_{off}) of the biotin-streptavidin bond is approximately 1/2500 days, 1/90 days, 1/1.4 days and 1/5 hours for T = -20, 4, 25 and 37 °C, respectively. These rates were calculated using energetics measurements from literature[5] and extrapolating these values using the Erying equation. These calculated k_{off} rates indicate that the bt-oligo-streptavidin conjugates as well as the antibody-DNA conjugates should be kept at -20 °C for long-term storage and at 4 °C until right before imaging.

Supplemental Note: Rationale for EGF stimulation time of 5 min.

The EGFR is known to rapidly internalize after stimulation with EGF. To minimize the impact of internalization on receptor levels, serum-starved cells were treated with EGF for five minutes prior to fixation. Other studies have shown that this is sufficient time to activate receptors as well as downstream kinase cascades to approximately peak levels, but not to promote significant endocytosis[6].

Supplemental Note: Fourier ring correlation.

FRC measures the integral imaging resolution based on spatial spectral similarity between two independent halves of the image[7]. In detail, we first split the fluorescence movie into small consecutive segments in time (100-200 frames for each segment), then combined even-numbered segments and odd-numbered segments separately to produce two super-resolution images. This ensures that (1) multiple localisations from the same blinking event are almost always grouped into one of the two images, avoiding fake spectral similarities created by consecutive localisations from the same blinking event, (2) long-term systematic effects such as microscope stage drift or thermal fluctuations are avoided in the calculation. The images were rendered with pixel size smaller than 1/5 of the estimated imaging resolution to avoid pixelation effect. Then we applied 2D Fast Fourier Transform (FFT) to both images and computed the correlation function between them for each ring of spectral components. Finally, we used the noise-based 2σ criterion to determine the cross-over point[7]. The resolution calculated by FRC is significantly larger than the resolution calculated by FWHM or DNFL (Supplemental Figure 7.) this is expected as the FRC resolution also incorporates spatial sampling frequency and labelling density.

Supplemental References

1 Niemeyer, C. M., Sano, T., Smith, C. L. & Cantor, C. R. Oligonucleotide-directed self-assembly of proteins: semisynthetic DNA--streptavidin hybrid molecules as connectors for the generation of macroscopic arrays and the construction of supramolecular bioconjugates. *Nucleic Acids Res.* **22**, 5530-5539 (1994).

2 Jones, M. L. & Kurzban, G. P. Noncooperativity of biotin binding to tetrameric streptavidin. *Biochemistry* **34**, 11750-11756 (1995).

3 Jungmann, R. *et al.* Multiplexed 3D cellular super-resolution imaging with DNA-PAINT and Exchange-PAINT. *Nat Methods* **11**, 313-318; DOI:10.1038/nmeth.2835 (2014).

4 Figueiredo, M. & Jain, A. K. Unsupervised learning of finite mixture models. . *IEEE Transactions on PAMI,* **24**, 381-396 (2002).

5 Chilkoti, A. & Stayton , P. S. Molecular Origins of the Slow Streptavidin-Biotin Dissociation Kinetics. *J. Am. Chem. SOC.,* **117**, 10622-10628 (1995).

6 Kleiman, L. B., Maiwald, T., Conzelmann, H., Lauffenburger, D. A. & Sorger, P. K. Rapid phospho-turnover by receptor tyrosine kinases impacts downstream signaling and drug binding. *Mol. Cell* **43**, 723-737; DOI:10.1016/j.molcel.2011.07.014 (2011).

7 Nieuwenhuizen, R. P. *et al.* Measuring image resolution in optical nanoscopy. *Nat Methods* **10**, 557-562; DOI: 10.1038/nmeth.2448 (2013).
